# The Quorum Sensing Inhibitor Hamamelitannin Increases Antibiotic Susceptibility of *Staphylococcus aureus* Biofilms by Affecting Peptidoglycan Biosynthesis and eDNA Release

**DOI:** 10.1038/srep20321

**Published:** 2016-02-01

**Authors:** Gilles Brackman, Koen Breyne, Riet De Rycke, Arno Vermote, Filip Van Nieuwerburgh, Evelyne Meyer, Serge Van Calenbergh, Tom Coenye

**Affiliations:** 1 Laboratory of Pharmaceutical Microbiology, Faculty of Pharmaceutical Sciences, Ghent University, Ghent, Belgium; 2 Laboratory of Biochemistry, Department of Pharmacology, Toxicology and Biochemistry, Faculty of Veterinary Medicine, Ghent University, Merelbeke, Belgium; 3 Inflammation Research Centre, VIB, Ghent, Belgium; 4 Department of Biomedical Molecular Biology, Ghent University, Ghent, Belgium; 5 Laboratory for Medicinal Chemistry, Faculty of Pharmaceutical Sciences, Ghent University, Ghent, Belgium; 6 Laboratory of Pharmaceutical Biotechnology, Faculty of Pharmaceutical Sciences, Ghent University, Ghent, Belgium

## Abstract

Treatment of *Staphylococcus aureus* infections has become increasingly challenging due to the rapid emergence and dissemination of methicillin-resistant strains. In addition, *S. aureus* reside within biofilms at the site of infection. Few novel antibacterial agents have been developed in recent years and their bacteriostatic or bactericidal activity results in selective pressure, inevitably inducing antimicrobial resistance. Consequently, innovative antimicrobials with other modes of action are urgently needed. One alternative approach is targeting the bacterial quorum sensing (QS) system. Hamamelitannin (2′,5-di-*O*-galloyl-d-hamamelose; HAM) was previously suggested to block QS through the TraP QS system and was shown to increase *S. aureus* biofilm susceptibility towards vancomycin (VAN) although mechanistic insights are still lacking. In the present study we provide evidence that HAM specifically affects *S. aureus* biofilm susceptibility through the TraP receptor by affecting cell wall synthesis and extracellular DNA release of *S. aureus*. We further provide evidence that HAM can increase the susceptibility of *S. aureus* biofilms towards different classes of antibiotics *in vitro*. Finally, we show that HAM increases the susceptibility of *S. aureus* to antibiotic treatment in *in vivo Caenorhabditis elegans* and mouse mammary gland infection models.

*Staphylococcus aureus* is an important causative agent of acute and chronic bacterial infections in humans and animals^1^. It is the leading cause of nosocomial infections worldwide and can cause a variety of infections, including skin and soft tissue infections, endocarditis, osteomyelitis and infections associated with medical devices^1^. Treatment of *S. aureus* infections has become increasingly challenging due to the rapid emergence and dissemination of methicillin-resistant strains (MRSA)^2^,^3^. In addition, *S. aureus* often reside within biofilms at the site of infection^4^. Biofilms are microbial sessile communities characterized by cells that are attached to a substratum or interface or to each other, are embedded in a self-produced matrix of extracellular polymeric substances and exhibit an altered phenotype compared to planktonic cells^5^. Within these biofilms*, S. aureus* displays enhanced resistance to antimicrobial agents^6^. This may be due to a decreased penetration of antibiotics, a decreased growth rate of the biofilm cells and/or a decreased metabolism of bacterial cells in biofilms^7^. In addition, the presence of persister cells and the expression of specific resistance genes in biofilms may contribute to this tolerance^8^.

Few novel antibacterial agents have been developed in recent years and their bacteriostatic or bactericidal activity results in selective pressure, with antimicrobial resistance as an inevitable consequence of their use^9^. For this reason, innovative antimicrobials with novel targets and modes of action are needed. One alternative approach is targeting the bacterial quorum sensing (QS) system. QS is a process by which bacteria produce and detect signal molecules and thereby coordinate their behaviour in a cell-density-dependent manner^10^. *S. aureus* uses at least two different QS systems to regulate their virulence, the *agr* system and the RAP/TRAP system^11^. Although the precise interplay between the two systems remains unclear, both are reported to alter gene expression through the control of RNAIII. In addition, *S. aureus* possesses a functional LuxS enzyme and produces AI-2, but does not possess a LuxPQ- or LsrB-type AI-2 receptor^12^,^13^. Given the role QS plays in the regulation of *S. aureus* pathogenicity, QS inhibitors (QSI) could be used as antipathogenic agents^11^,^14^,^15^. Several inhibitors targeting the QS system of *S. aureus* have been described, but their mechanism of action mostly remains unknown^11^. Hamamelitannin (2′,5-di-*O*-galloyl-d-hamamelose; HAM) was previously suggested to block QS through the TraP QS system^16^ and was shown to increase *S. aureus* biofilm susceptibility towards vancomycin (VAN) although mechanistic insights are still lacking^17^.

In the present study we provide evidence that HAM affects *S. aureus* biofilm susceptibility through the TraP receptor, resulting in altered cell wall synthesis and extracellular DNA (eDNA) release. We further provide evidence that HAM can increase the susceptibility of *S. aureus* biofilms towards different classes of antibiotics. Finally, HAM is capable of increasing the susceptibility of *S. aureus* towards antibiotics in *Caenorhabditis elegans* and mouse mammary gland infection models.

## Results

### HAM affects *S. aureus* susceptibility to various classes of antibiotics

**Figure 1:**
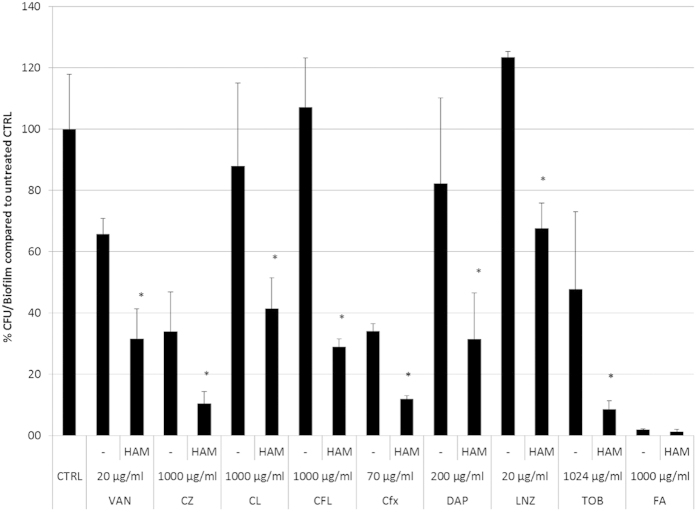
Effect of HAM on biofilm susceptibility of *S. aureus* Mu50 against different types of antibiotics. The percentage CFU/biofilm ± s.d. (compared to untreated control biofilm) for biofilms exposed to vancomycin (VAN), cefazolin (CZ), cefalonium (CL), cephalexin (CFL), cefoxitin (Cfx), daptomycin (DAP), linezolid (LNZ), tobramycin (TOB) or fusidic acid (FA) alone or in combination with HAM. *significantly increased killing was observed when biofilms were treated with the combination of the antibiotic and HAM compared to treatment with the antibiotic alone (*n* ≥ 9; one-way; p < 0.05).

We evaluated the effect of HAM on susceptibility of *S. aureus* towards a wide range of antibiotics. These included cefazolin (CZ), cefalonium (CL), cephalexin (CFL), cefoxitin (Cfx), daptomycin (DAP), linezolid (LNZ), tobramycin (TOB) and fusidic acid (FA). HAM had no effect on the MIC of these antibiotics against *S. aureus* Mu50 (Supplementary Table S1). Although minor differences in MIC were observed for some antibiotics, these differences were within the acceptable margin of error and were not considered as relevant. As such FIC indices were ≥0.5 for all combinations indicating that there was no synergistic activity and that the interactions observed are indifferent. In contrast, significantly increased killing of *S. aureus* Mu50 biofilm cells was observed when CZ, CL, CFL, Cfx, DAP, LNZ and TOB were used in combination with HAM (Fig. 1). Increased killing of biofilms cells by antibiotics used in combination with HAM was also observed for other *S. aureus* strains (Supplementary Figure S1).

### HAM affects *S. aureus* biofilm susceptibility by interfering with QS

**Figure 2:**
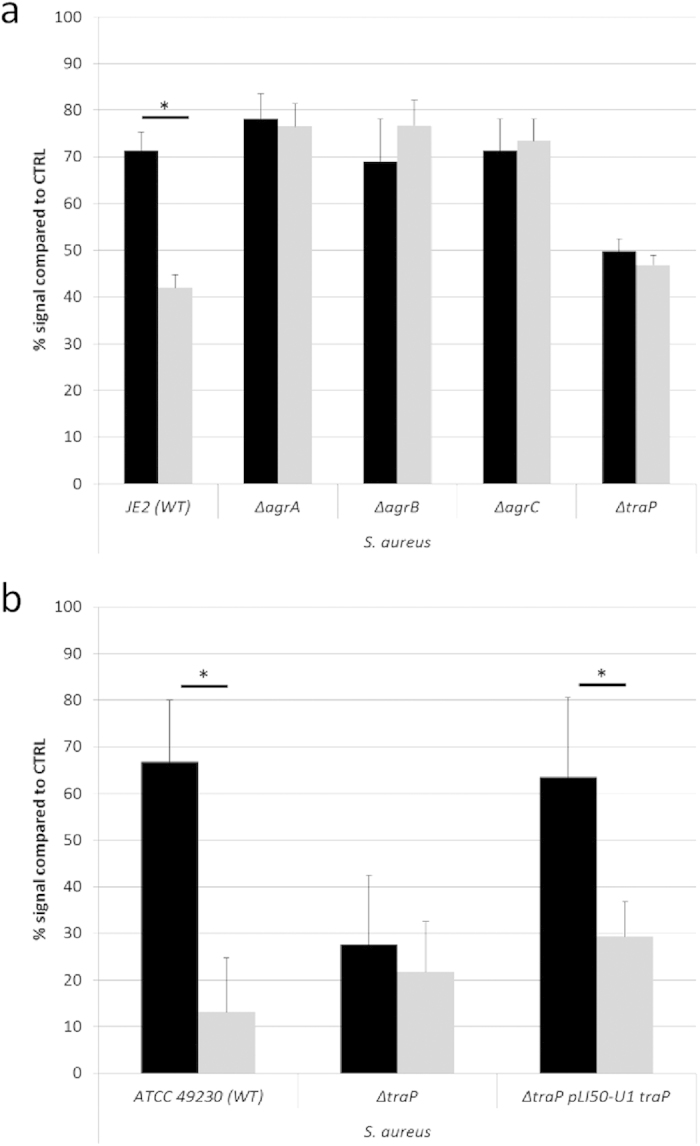
Effect of HAM on biofilm susceptibility of *S. aureus* WT strains, QS mutants and complemented strains. Biofilms of *S. aureus* JE2 WT and QS mutants (**a**) and *S. aureus* ATCC 49230, Δ*traP* mutant and the *traP* complemented strain Δ*traP pLI50-U1 traP* (**b**) were exposed to VAN alone (black bars) or a combination of HAM and VAN (grey bars). Cell viability was quantified by CTB staining and signals are presented as percentages (average ± s.d.) compared to the signal of an untreated biofilm. *significantly different signals were observed between both treatments (*n* ≥ 9; one-way; p < 0.01).

We evaluated the effect of HAM on VAN susceptibility of *S. aureus* strains with mutations in the QS system (Δ*agrA,* Δ*agrB*, Δ*agrC*, Δ*traP* and Δ*luxS*). In addition, we evaluated the effect of HAM on VAN susceptibility of *S. aureus* strains with mutations in other regulatory genes, or in genes known to affect biofilm formation and/or resistance (e.g. Δ*sarA*, Δ*sarU*, Δ*icaA*, Δ*kpdD*, Δ*codY*). HAM was used in concentrations well below the MIC, had no effect on growth and had no effect on the number of metabolically active cells when tested against biofilms formed by the *S. aureus* mutants (Supplementary Figure S2). In contrast, when VAN was used in combination with HAM, the *S. aureus* JE2 and ATCC 49230 wild-type strains became more susceptible to VAN (Fig. 2). However, this increased susceptibility to VAN in the presence of HAM disappeared in *S. aureus* strains lacking a functional *agr* or *traP* QS system (Fig. 2). In addition, the Δ*traP* mutant was more susceptible to VAN, even in the absence of HAM. Importantly, the complemented Δ*traP* mutant strain Δ*traP pLI50-U1 traP* displayed decreased susceptibility towards VAN alone in comparison to the Δ*traP* mutant strain. In addition, the susceptibility of the complemented strain towards VAN was increased when VAN was used in combination with HAM (Fig. 2). Mutations in other genes did not affect the activity of HAM (Supplementary Figure S2).

**Figure 3:**
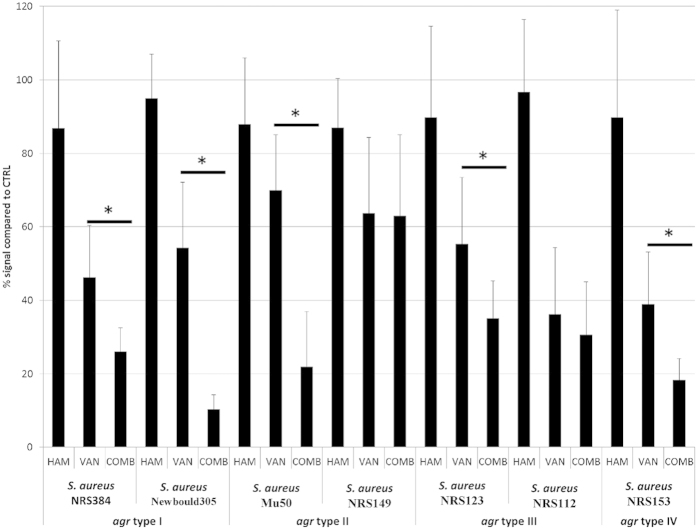
Effect of HAM on biofilm susceptibility of *S. aureus* strains belonging to different *agr* groups. Biofilms were exposed to HAM or VAN alone or a combination of VAN and HAM (COMB). Cell viability was quantified by CTB staining and signals are presented as percentages (average ± s.d.) compared to the signal of an untreated biofilm. *significantly different signals were observed between cells exposed to VAN alone and exposed to a combination of VAN and HAM (*n* ≥ 9; one-way; p < 0.01).

*S. aureus* strains belong to one of four *agr* groups depending on the amino acid sequence and length of the AIP and the cognate receptor AgrC^18^. As our results indicate that the *agr* QS system also plays a role in the activity of HAM, we evaluated whether HAM was active against *S. aureus* strains belonging to different *agr* groups. Although HAM had no effect by itself and although the susceptibility of the biofilms of *S. aureus* strains belonging to different *agr* groups towards VAN differed, in most cases more biofilm cells were killed when strains were treated with a combination of VAN and HAM compared to VAN treatment alone (Fig. 3). However, no increased susceptibility was observed for *S. aureus* NRS149 (*agr* group II) and *S. aureus* NRS112 (*agr* group III) (Fig. 3). Although the latter strain is reported to contain a mutation in *agr*, resulting in an already decreased RNAIII production, no mutations in the QS system have been reported for *S. aureus* NRS149. This indicates that HAM is active against *S. aureus* strains with different *agr* types, although its activity may be strain-dependent.

HAM did not affect susceptibility of closely related *Staphylococcus* species (Supplementary Figure S3) or that of selected Gram negative bacteria (Supplementary Figure S4) indicating that the effect of HAM on biofilm susceptibility is specific for *S. aureus*.

### HAM affects biofilm susceptibility by affecting peptidoglycan synthesis and cell wall thickness

Next, we wanted to elucidate the mechanism by which HAM increases the susceptibility of *S. aureus* biofilm towards VAN. HAM had no effect on growth of *S. aureus* Mu50, nor did it affect membrane integrity of *S. aureus* biofilm cells after 10 min or 24 h of incubation (Supplementary Figure S5).

**Figure 4:**
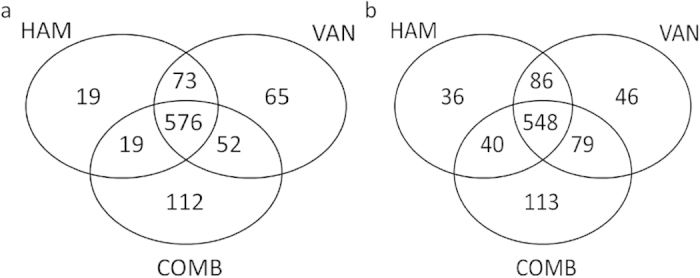
Number of differentially expressed genes in treated cells compared to untreated cells. Number of genes differentially (**a**) up- or (**b**) downregulated in *S. aureus* Mu50 biofilm cells exposed to HAM, VAN or a combination of HAM and VAN compared to unexposed cells.

To further pursue the molecular mechanism by which HAM affects biofilm susceptibility towards VAN, we compared the transcriptome of treated versus untreated *S. aureus* Mu50 biofilm cells using RNA sequencing. Treatment with either HAM or VAN alone or treatment with a combination of both had a significant impact on gene-expression (Fig. 4 and supplementary Figure S6). Between 600 and 800 genes were differentially up- or down-regulated after treatment with HAM, VAN or a combination of both (Supplementary Table S2).

Treatment with HAM (either alone or in combination with VAN) resulted in a significant downregulation of the *traP* gene compared to the untreated control (−1.54 and −1.84 fold change for HAM and combination of VAN and HAM, respectively). Although treatment with HAM resulted in an up-regulation of *agrA* and *agrC* (1.56 and 1.69 fold change, respectively), no difference in expression of *agr* genes was observed with the combination treatment.

Not unexpectedly, several genes that were previously reported to be differentially expressed in glycopeptide resistant strains or after glycopeptide treatment were differentially expressed after VAN treatment in the present study as well (Supplementary Table S3)^19^,^20^,^21^,^22^,^23^,^24^,^25^. An important response mechanism of *S. aureus* towards glycopeptides is an increased cell wall synthesis and increased cell wall thickness^26^.

To achieve this, *S. aureus* cells upregulate uptake of building blocks, upregulate peptidoglycan synthesis and upregulate the biosynthesis of precursors^27^. This response was also observed in the VAN treated biofilms and in biofilm cells treated with a combination of VAN and HAM (Supplementary Figure S6).

Moreover, SAV1422, encoding a glucose specific enzyme was 1.80 and 3.68 fold upregulated in the presence of VAN alone or in combination with HAM. Similarly, several genes including *sgtA*, *sgtB* and *pbp2-4* which are related to cell wall biosynthesis were upregulated in the presence of VAN (either alone or in combination with HAM) (Supplementary Figure S6)^28^. These genes are directly involved in peptidoglycan (precursor) biosynthesis^28^. This indicates that when VAN is added (alone or in combination with HAM), more glucose will be taken up by the cell and synthesis of peptidoglycan will be increased.

Although HAM alone does not change the expression of the above-mentioned genes, several genes involved in biosynthesis of precursors of peptidoglycan components are differentially expressed when HAM was used in combination with VAN compared to exposure to VAN alone. For example *glmS*, *asd, dapB* and *dapD* were significantly downregulated in cells exposed to HAM in combination with VAN while no difference in regulation was observed in cells exposed to VAN alone (Supplementary Table S2). In addition, *glnA, lysC* and *dapA* were upregulated and *narH* and *narG* were downregulated in cells exposed to VAN, while no significant change in expression were observed in cells exposed to VAN in combination with HAM compared to the untreated cells. In addition, several genes were strongly down-regulated in cells treated with a combination of VAN and HAM compared to VAN treatment alone, e.g. *hutU* (−9.6 compared to −4.7 fold change), *hutI* (−12.2 compared to −5.6 fold change), *gltD* (−26.1 compared to −3.4 fold change) and *gltB* (−15.4 compared to −2.6 fold change) (supplementary Figure S6). These genes are all involved in biosynthetic pathways leading to the synthesis of precursors of peptidoglycan and or glutamine consuming pathways.

**Figure 5:**
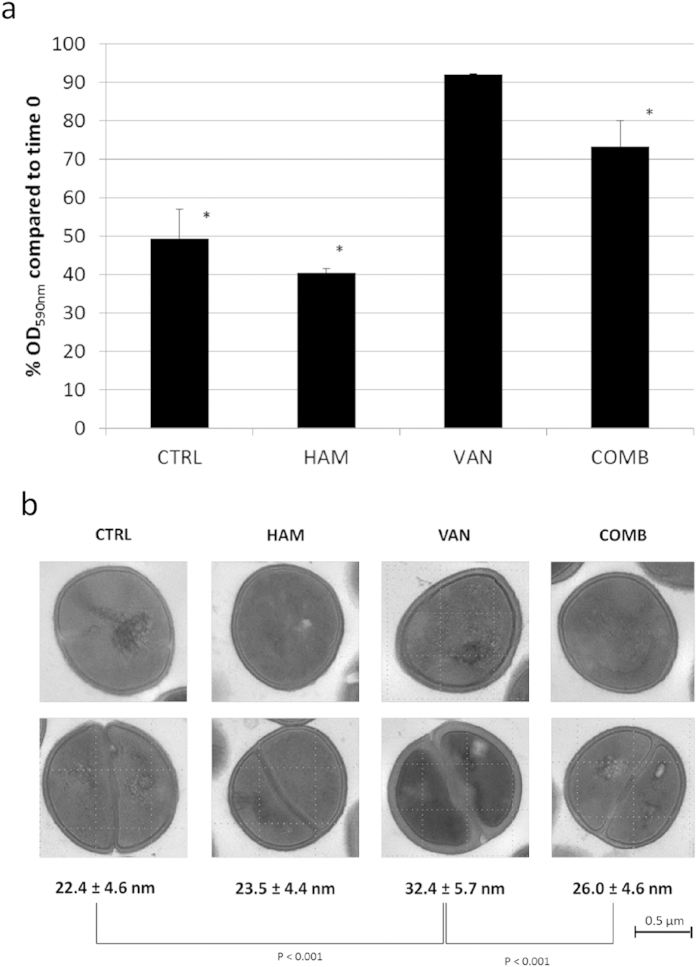
Effect of treatment on lysostaphin susceptibility and thickness of the cell wall. (**a**) OD590 nm (average ± s.d.) after 10 min lysostaphin treatment of *S. aureus* Mu50 biofilm cells receiving no pre-treatment (CTRL) or a pre-treatment with HAM, VAN or a combination of HAM and VAN (COMB). The OD590nm after 10 min was compared to the OD590 before the addition of lysostaphin (set at 100%).*the percentage OD590 nm is significantly different from that of the cells receiving pre-treatment with VAN (*n* ≥ 6; one-way; p < 0.05). (**b**) Changes in cell wall thickness of *S. aureus* Mu50 biofilm cells receiving no treatment (CTRL) or a treatment with HAM, VAN or a combination of HAM and VAN (COMB). Significant (*n* ≥ 100 cells; one way; p < 0.001) differences in cell wall thickness were observed between the untreated and VAN treated biofilm cells and between biofilm cells receiving a treatment with VAN or a combination of VAN and HAM.

Combined, these RNA-sequencing data point towards changes in peptidoglycan biosynthesis after treatment with VAN alone or VAN in combination with HAM. To confirm these changes, we evaluated the susceptibility of biofilm cells treated with HAM, VAN or a combination of VAN and HAM towards lysostaphin and measured cell wall thickness by electron microscopy (Fig. 5). The untreated biofilm cells or those treated with HAM were sensitive to lysostaphin while biofilm cells receiving a pre-treatment with VAN were much more resistant to lysostaphin treatment (Fig. 5A). In contrast, biofilm cells treated with VAN in combination with HAM displayed a significant higher sensitivity towards lysostaphin compared to those treated with VAN alone (Fig. 5A). This suggests that changes in cell wall thickness occur after treatment. In order to obtain direct evidence we evaluated the effect of VAN, HAM or a combination of HAM and VAN on cell wall thickness by electron microscopy (Fig. 5B). The *S. aureus* Mu50 biofilm cells treated with VAN had significant thicker cell walls compared to untreated biofilm cells (Fig. 5B). Although HAM alone did not affect cell wall thickness, significant thinner cell walls were measured for cells treated with a combination of VAN and HAM compared to VAN treatment alone (Fig. 5B).

### HAM affects biofilm susceptibility by altering the amount of eDNA in the biofilm matrix

**Figure 6:**
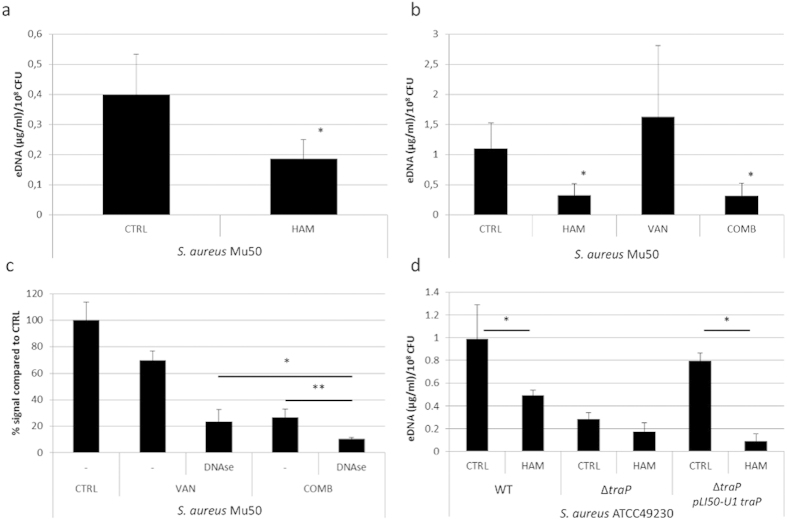
HAM alters the amount of eDNA in the biofilm and thereby affects biofilm susceptibility. (**a**) The amount of eDNA (average ± s.d.) present in *S. aureus* Mu50 biofilms formed in the absence (CTRL) or presence of HAM. (**b**) The amount of eDNA (average ± s.d.) in 24 h old biofilms receiving no treatment (CTRL) or a treatment with HAM or VAN alone or a combination of VAN and HAM (COMB). (**c**) Cell viability as quantified by CTB staining for *S. aureus* Mu50 biofilms receiving no pre-treatment, or a pre-treatment with DNase followed by (i) no treatment (CTRL), (ii) a treatment with VAN or (iii) a combination of VAN and HAM (COMB). CTB signals are presented as percentages (average ± s.d.) compared to the signal of an untreated biofilm. (**d**) The amount of eDNA (average ± s.d.) present in biofilms of *S. aureus* ATCC 49230, Δ*traP* mutant and the *traP* complemented strain Δ*traP pLI50-U1 traP* formed in the absence (CTRL) or presence of HAM. *indicates significant (p < 0.05) differences between the amount of eDNA present in the treated biofilms compared to the CTRL biofilm (**a**,**b**,**d**) or in % signal compared to the biofilms treated with VAN (**c**). **significant (p < 0.05) differences in percentage signal are observed between biofilm cells treated with a combination of VAN and HAM following no pre-treatment or a pre-treatment with DNase.

Our RNA sequencing data also indicated that the *lrgAB* genes were differentially expressed after treatment. These genes were significantly downregulated after treatment with VAN (−3.09 and −2.44 fold change, respectively) while no difference in expression was observed after combination treatment (Supplementary Table S2). The *lrgAB* genes were previously reported to affect autolysis and eDNA release^29^. Also, the expression of SAV0913 (encoding the autolysin *N*-acetylmuramyl-L-alanine amidase), *lytM* (encoding the glycyl-glycine endopeptidase) and SAV1051 (encoding the autolysin transcription regulator) differed between treatment with VAN alone or in combination with HAM (Supplementary Table S2). This led us to the hypothesis that HAM alters the amount of eDNA in the biofilm matrix and thereby alters susceptibility of the biofilm. To confirm this hypothesis we evaluated the amount of eDNA in *S. aureus* biofilms formed in the presence of HAM (Fig. 6A). In addition, we evaluated whether exposure to HAM would alter eDNA concentrations in an already established biofilm (Fig. 6B). Less eDNA was present in the matrix under both conditions indicating that HAM indeed alters the amount of eDNA present in the biofilm. Also, significantly less eDNA was present in biofilms exposed to a combination of VAN and HAM (Fig. 6B). To confirm the role of eDNA in biofilm susceptibility, we evaluated the effect of DNAse pre-treatment on susceptibility toward VAN. Significantly less metabolically active cells were present in VAN treated biofilms when these biofilms were pre-treated with DNase (reduction of 30.4 ± 9.8% and 76.3 ± 6.2% compared to the untreated control after VAN treatment of biofilm receiving no pre-treatment or a pre-treatment with DNase, respectively) (Fig. 6C). In addition, a significant reduction in metabolically active cells was also observed when the combination treatment was preceded by DNase treatment (reduction of 73.1 ± 3.7% and 89.4 ± 1.1% after combination treatment of biofilms receiving no pre-treatment or a pre-treatment with DNase, respectively) (Fig. 6C).

Finally, to confirm that TraP is the target of HAM, we measured the amount of eDNA present in a *S. aureus* ATCC 49230 Δ*traP* mutant and evaluated whether HAM altered eDNA production in this mutant. Significantly less eDNA was present in the biofilm matrix of the Δ*traP* mutant strain compared to the WT strain (Fig. 6D). No difference in the amount of eDNA was observed when Δ*traP* mutants formed biofilms in the presence of HAM (Fig. 6D). Similar results were also obtained for the *S. aureus* JE ∆*traP* mutant strain (data not shown). In addition, eDNA production of the complemented strain was increased compared to the Δ*traP* mutant when no HAM was used and eDNA production were reduced to the levels of eDNA production of the Δ*traP* mutant when HAM was present during biofilm formation. This suggests that HAM exerts this effect on eDNA production by interfering with the TraP receptor. Interestingly, although no difference in eDNA was observed between WT and Δ*agrA* biofilms, more eDNA was present in the Δ*agrB* and Δ*agrC* mutant compared to the WT strain (2.66 ± 0.58, 3.21 ± 0.75 and 1.20 ± 0.21 μg eDNA/ml/10^8^ CFU, for Δ*agrB*, Δ*agrC* and WT biofilms, respectively).

Although an upregulation of the *mecA* gene was observed after treatment with HAM, VAN or both, several other genes involved in resistance towards different types of antibiotics were observed to be downregulated in biofilms after treatment. These included *aadD*, *fmtC*, *norA*, *tcaB*, SAV0690 (*tetR*) and genes encoding beta-lactamases (SAV1504 and SAV1815) and multidrug resistance proteins (SAV0726, SAV1761, SAV2462). Downregulation of the expression of these genes might, in addition to the above mentioned mechanisms, also explain why HAM increases susceptibility towards various classes of antibiotics.

### HAM represses antibiotic induced increase in bacterial virulence

**Figure 7:**
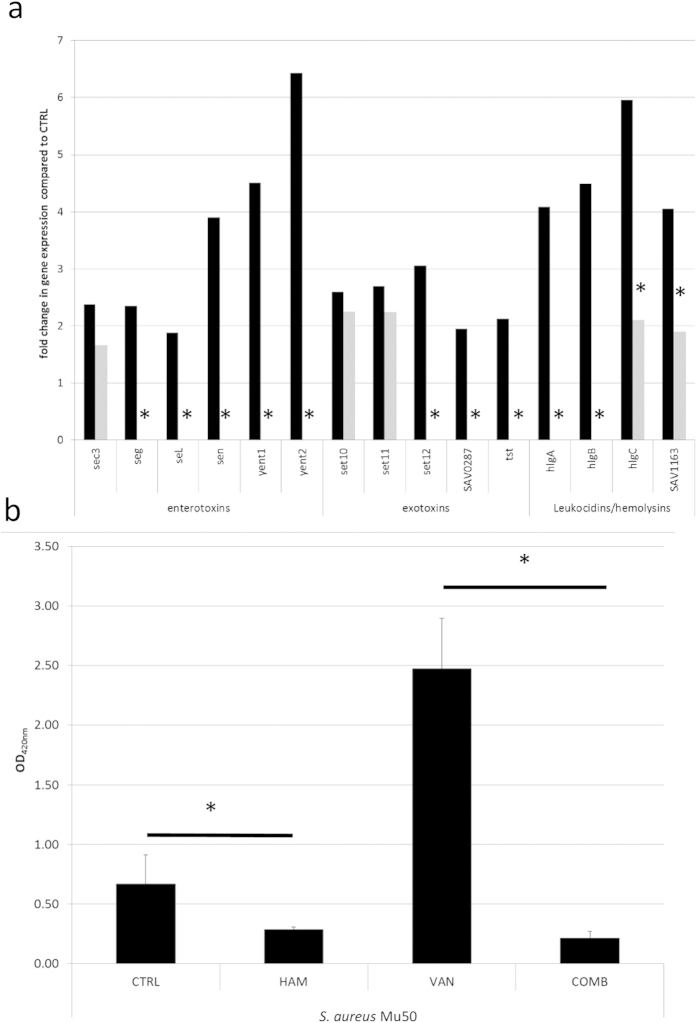
Effect of treatment on expression of virulence genes and hemolytic activity. (**a**) Fold changes in expression of selection of virulence genes in *S. aureus* Mu50 biofilms treated with VAN (black bars) or a combination of VAN and HAM (grey bars) compared to expression in untreated biofilm cells. *significant (p < 0.01) differences are observed in gene expression between biofilm cells treated with VAN or a combination of VAN and HAM. (**b**) OD420 nm (average ± s.e.m.) (as indication of hemolytic activity) of rabbit blood incubated with the supernatant of *S. aureus* Mu50 untreated biofilm cells (CTRL) or biofilm cells treated with HAM or VAN alone or a combination of VAN and HAM (COMB). *significant (p < 0.01) differences in OD420 nm between biofilm cells treated with VAN or a combination of VAN and HAM and between untreated biofilms and HAM treated biofilms.

Our RNA sequencing data showed that VAN treatment resulted in an upregulation of several genes encoding enterotoxins (e.g. *seg*, *sen*, *yent1* and *yent2*), exotoxins (*set10, set11, set12* and toxic shock toxin *tst*) and leukocidins/hemolysins (e.g. *hlgA*, *hlgB*, *hlgC* and SAV1163) (Fig. 7A). An increase in virulence gene expression as a response to antibiotics was previously also observed in other studies^30^,^31^,^32^. In contrast, when VAN was used in combination with HAM, no upregulation was observed for most of these genes (Fig. 7A). In addition, although an upregulation was observed for *sec3*, *hlgC* and SAV1163 in cells treated with a combination of VAN and HAM (fold change of 1.67, 2.11 and 1.90 compared to no treatment, respectively), this upregulation was significantly lower than the upregulation observed in cells treated with VAN alone (fold change of 2.38, 5.96 and 4.05 compared to vehicle control, respectively) (Fig. 7A). In line with these observations we also observed reduced α-hemolysin activity in supernatants of *S. aureus* Mu50 biofilms treated with HAM, while an increased hemolytic activity was observed for biofilms treated with VAN (Fig. 7B). In contrast, the supernatant of biofilm cells treated with a combination of VAN and HAM showed significantly reduced hemolytic activity (absorbance of 0.22 ± 0.06, 2.47 ± 0.42 and 0.67 ± 0.24 for supernatants from biofilms treated with a combination of VAN and HAM, VAN alone or untreated cells) (Fig. 7B).

### HAM affects biofilm susceptibility and virulence *in vivo* in *C. elegans* and in mouse mammary gland infection models

**Figure 8:**
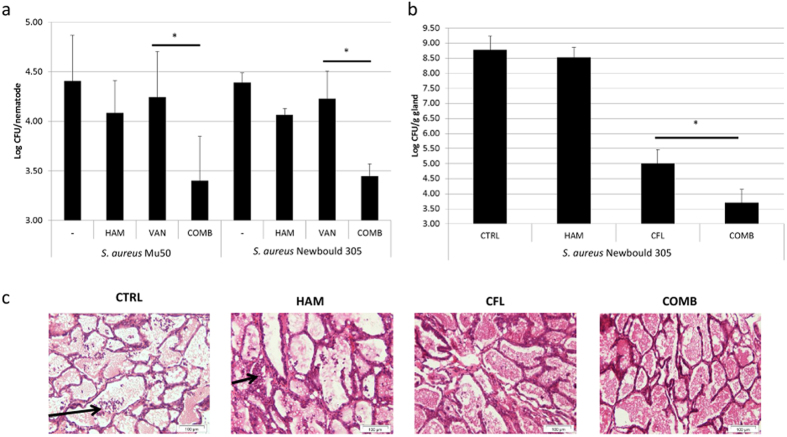
Effect of treatment on *in vivo* susceptibility of *S. aureus*. (**a**) Log CFU/nematode (average ± s.d.) in *C. elegans* nematodes infected with *S. aureus* Mu50 or Newbould 305, receiving no treatment or a treatment with HAM, VAN or a combination of VAN and HAM (COMB). (**b**) Log CFU/g mammary gland (average ± s.d.) of mice infected with *S. aureus* Newbould305 receiving no treatment or a treatment with HAM, CFL or a combination of CFL and HAM (COMB). (**c**) Histological evaluation of mammary glands of mice infected with *S. aureus* Newbould305 receiving no treatment or a treatment with HAM, CFL or a combination of CFL and HAM (COMB). Arrows indicate influx of neutrophils. *significant differences in log CFU/nematode (**a**) or log CFU/g mammary gland (**b**) between the indicated samples (p < 0.01).

Although treatment with HAM and VAN alone resulted in an increased survival of infected *C. elegans* nematodes (Supplementary Figure S7), either HAM or VAN alone had no effect on the number of bacteria present in *C. elegans* nematodes after 24 h of infection (Fig. 8A). In contrast, significantly less CFU/nematode were present when a combination of HAM and VAN was used to treat the infected nematodes (Fig. 8A). In addition, a significant higher percentage of *C. elegans* nematodes survived infection after treatment with a combination of HAM and VAN compared to treatment with VAN alone (Supplementary Figure S7).

Next, we evaluated the effect of HAM on *S. aureus* susceptibility in an established murine mammary gland infection model with the bovine mastitis isolate *S. aureus* Newbould 305^33^. Bovine mastitis caused by *Staphylococci* is typically treated with cephalosporins and as such CFL was used^33^.

As for *C. elegans*, HAM itself had no effect on the number of CFU present in the infected mouse mammary glands (Fig. 8B). Although treatment with CFL resulted in a decrease in the number of CFU/g mammary gland when used alone, significantly less CFU was present after treatment with CFL in combination with HAM (Fig. 8B). Macroscopical signs of inflammation were mainly observed in the glands of mice receiving no treatment and to a much lesser extent in the glands of mice receiving a treatment with HAM or CFL (Supplementary Figure S8). In addition, an influx of neutrophils was observed in the alveoli of the glands in the untreated conditions or the mice receiving treatment with HAM alone (Fig. 8C). This innate immune response was not observed in the mice receiving treatment with CFL or a combination of CFL and HAM (Fig. 8C).

## Discussion

HAM was discovered via virtual screening of a library of small molecules using a pharmacophore model based on RNAIII inhibiting peptide (RIP), a peptide that blocks QS in *S. aureus*^16^. It was therefore suggested that HAM also blocks QS through the TraP QS system^16^. However, its target was never validated and as such it is still unclear whether HAM truly affects QS through the TraP receptor. In addition, although HAM was shown to increase *S. aureus* biofilm susceptibility towards vancomycin^17^, several questions remained unanswered: how does HAM affect *S. aureus* susceptibility at the molecular level? Does this effect also occur in other closely or unrelated species and with other antibiotics? Finally, the potential clinical application of HAM had not been evaluated in relevant *in vivo* models of *S. aureus* infection.

First, we investigated the spectrum of HAM. Our results show that HAM increases the susceptibility of *S. aureus* biofilms to different classes of antibiotics, indicating that the effect of HAM is not limited to combinations with VAN alone. Although HAM was previously shown to affect attachment and biofilm formation of *S. epidermidis* and *Acinetobacter baumanii*^34^,^35^,^36^ our result show that HAM only affected susceptibility of *S. aureus* and not that of unrelated Gram negative or more closely related Gram positive bacteria.

Secondly, we addressed the specificity of HAM for the TraP receptor in the *S. aureus* QS system employing two strategies. First we evaluated the effect of HAM on the susceptibility of biofilms of *S. aureus* strains with mutations in genes directly involved in the QS systems or in genes involved in biofilm formation and virulence. Secondly, we identified genes that were differentially expressed upon treatment, using RNA sequencing. HAM had no effect on biofilm susceptibility of *S. aureus* QS mutants while the effect was maintained for strains with mutations in other genes. This indicates that HAM truly affects biofilm susceptibility through the *S. aureus* QS system. Although our results suggest that the *agr* QS system is also involved in this mechanism of action, we do believe that the TraP receptor might be the first target of HAM for several reasons. First, no additional effect on biofilm susceptibility is observed for the combination therapy in *agr* mutant strains, while these *agr* mutants are as susceptible towards VAN as the WT strain. In contrast, the Δ*traP* mutant is much more susceptible towards VAN. Secondly, we can exclude the possibility that AgrC (i.e. the receptor for AIP in the *agr* QS system) will be the target of HAM since HAM exerted its effect in strains belonging to different *agr* groups. Although this does not exclude the possibility that HAM interacts with AgrA, a downstream regulator of the *agr* QS system we believe this is unlikely. HAM fits the pharmacophore model of RIP, a peptide described to block QS by affecting TraP^16^, while it is structurally unrelated to savarin, a specific inhibitor of AgrA^37^. Thirdly, the *traP* gene was downregulated in *S. aureus* Mu50 treated with HAM alone or in combination with VAN, while expression of *agrA* and *agrC* was only affected when HAM was used alone. Together, these data indicate that HAM affects biofilm susceptibility through TraP.

Next, we addressed the question on how HAM resulted in an increased susceptibility towards antibiotics. *S. aureus* Mu50 does not contain the *vanAB* genes that play a role in VAN resistance in enterococci, but multiple genes contribute to glycopeptide resistance in this (and other) *S. aureus* strain^26^,^27^. Our combined data indicate that HAM affects a set of genes leading to changes in cell wall thickness and amount of eDNA in the biofilm matrix.

The molecular mechanism behind the increased cell wall thickness is the lower expression of several genes involved in the biosynthesis of peptidoglycan precursors such as e.g. *glmS, lysC*, *asd*, *dapA* in the presence of a combination of VAN and HAM, compared to exposure to VAN alone. GlmS and enzymes encoded by the *dap* operon play a role in the production of GlcN-6-P and in the conversion of L-aspartate to L-lysine, respectively. Both GlcN-6-P and L-lysine are important components of the cell wall. In addition, the expression of genes belonging to pathways leading to L-glutamate production (from L-histidine or α-ketoglutarate), to the glutamine consuming pyrimidine pathway, and genes from the *nas* operon were also differentially expressed in the presence of a combination of VAN and HAM, compared to exposure to VAN alone. Combined, these differences are likely to result in depletion of glutamine during VAN treatment, a depletion that will not be observed when VAN is combined with HAM, and this glutamine depletion can affect susceptibility of *S. aureus* towards VAN^27^.

Although genes involved in final stages of peptidoglycan synthesis are also upregulated in *S. aureus* Mu50 biofilm cells exposed to a combination of VAN and HAM (including *sgtA*, *sgtB* and *pbp2-4*), this will not lead to increased cell wall thickness as HAM will prevent *S. aureus* cells from increasing the required peptidoglycan precursor synthesis.

Our results further indicate that the release of eDNA plays an important role in the mechanisms of action of HAM. Significantly less eDNA was present in the biofilm matrix of biofilms treated with HAM or in the Δ*traP* mutant, and HAM had no effect on the amount of eDNA in the matrix of the biofilm of the Δ*traP* mutant. These changes could also be complemented. Removal of eDNA had a significant impact on biofilm susceptibility indicating that changes in eDNA as a consequence of HAM-treatment will affect biofilm susceptibility. This is in agreement with the observation that eDNA is a major structural adhesin in *S. aureus* biofilms^38^,^39^,^40^ and that eDNA can directly bind VAN thereby trapping it before it can reach the cell^41^.

Both the increased cell wall thickness and presence of eDNA lead to trapping of more VAN, decreasing the VAN diffusion constant and eventually decreasing susceptibility towards VAN^27^. HAM affects both response mechanisms, thereby making *S. aureus* more susceptible to antibiotic treatment. Changes in peptidoglycan structure and the amount of eDNA in the biofilm matrix are likely to also affect the susceptibility towards other antibiotics including but not limited to β-lactam antibiotics and daptomycin^41^,^42^,^43^ and this was confirmed in the present study.

Although QS systems are activated and QS-regulated phenotypes are apparent *in vitro* (in the absence of a host), it is unclear whether QS requires a host to be fully activated. QS-based regulation of different phenotypes depends on the environment in which the bacteria reside, and in addition, many chemical compounds, such as QS inhibitors are prone to chemical changes or degradation under *in vivo* conditions, thereby making them less active. This indicates that the host can play a role in the way the QS system is activated and in whether the compound displays *in vivo* activity (or not). For this reason, we evaluated the effect of HAM on *S. aureus* susceptibility in a murine model of *S. aureus* mastitis infection. *S. aureus* is one of the most important etiological agents of subclinical and clinical mastitis^44^,^45^. In addition, once established, *S. aureus* infections are extremely hard to eradicate from the mammary gland^33^. We show that although HAM by itself did not result in a significant decrease in cell numbers, HAM increases susceptibility of *S. aureus* towards CFL in a murine model of mastitis infection.

Altogether our findings demonstrate that HAM interferes with QS in *S. aureus* and thereby increases susceptibility of *S. aureus* biofilms to various antibiotics. Data from animal experiments suggest that HAM has the potential to increase the effect of antibiotics *in vivo* and could be used in combination treatment schemes.

## Materials and Methods

### Reagents used

Hamamelitannin (HAM), vancomycin (VAN), cefoxitin (CFX), cefazolin (CZ), cefalonium (CL), cephalexin (CFL), linezolid (LNZ) and fusidic acid (FA) were purchased from Sigma Aldrich (Bornem, Belgium). Tobramycin (TOB) and daptomycin (DAP) were purchased from TCI (Tokyo, Japan). HAM was stored in DMSO at −20 °C. All antibiotics were dissolved in ultrapure water (with the exception of CZ, CL and CFL, which were dissolved in phosphate buffer). HAM was used at 250 μM concentrations unless otherwise mentioned.

### Bacterial strains and growth conditions

The following strains were used as previously described: methicillin-resistant *Staphylococcus aureus* Mu50 (MRSA Mu50), *Pseudomonas aeruginosa* PAO1, *Burkholderia cenocepacia* LMG16656^17^, *Staphylococcus capitis* ET005, *Staphylococcus caprae* NW003, *Staphylococcus cohnii* ET027, *Staphylococcus chromogenes* NW110, *Staphylococcus epidermidis* ET013 and ET041, *Staphylococcus haemolyticus* ET070, *Staphylococcus hyicus* ET053, *Staphylococcus hominis* ET056, *Staphylococcus lentus* ET004, *Staphylococcus lugdunensis* NW045, *Staphylococcus saprophyticus* ET094, *Staphylococcus pasteuri* ET054, *Staphylococcus schleiferi* NW139, *Staphylococcus warneri* ET019 and *Staphylococcus xylosus* ET018^46^, and *S. aureus* Newbould 305 (ATCC 29740)^33^. *S. aureus* NRS384 (*agr* I), NRS149 (*agr* II), NRS123 (*agr* III), NRS112 (*agr* III), NRS153 (*agr* IV), JE2 (MRSA USA300; *agr* I) and transposon mutants NE1532 (Δ*agrA*), NE95 (Δ*agrB*), NE873 (Δ*agrC*), NE294 (Δ*traP*), NE1746 (Δ*luxS*), NE423 (Δ*kdpD*), NE1249 (Δ*kdpE*), NE1193 (Δ*sarA*), NE96 (Δ*sarU*), NE1555 (Δ*codY*), NE1474 (Δ*cshA*), NE1241 (Δ*nuc*), NE37 (Δ*icaA*) and NE13 (Δ*rbsU*) were obtained through the Network on Antimicrobial Resistance in *Staphylococcus* (NARSA) (distributed by BEI Resources, NIAID, NIH). *S. aureus* ATCC 49230, Δ*traP* mutant and the *traP* complemented strain Δ*traP pLI50-U1 traP* were kindly provided by Dr. Smeltzer and Dr. Beenken^47^. All strains were cultured in Mueller-Hinton broth (MH, Oxoid, Basingstoke, England) at 37 °C under aerobic conditions. All mutants were grown in the presence of appropriate amounts of erythromycin.

### Determination of the MIC of HAM and effect of HAM on growth

MICs of HAM against the different strains used were determined in triplicate using flat-bottom 96-well microtiter plates (TPP, Trasadingen, Switzerland) as previously described^17^. If the MIC determined in both conditions differed, checkerboard testing^48^ was performed to determine whether the interaction is synergistic (fractional inhibitory concentration [FIC] index ≤0.5) or indifferent (FIC index >0.5). In addition, the effect on growth was investigated as follows. Overnight cultures of the different strains were diluted to approximately 5 × 10^7^ colony-forming units (CFU)/ml in MH. Ten μl of this suspension was added to the wells of a 24-well microtiter plate and mixed with 990 μl MH (in the presence or absence of HAM). Plates were incubated at 37 °C and the OD_590 nm_ was measured every 30 min for 24 h. Three wells were included per condition and the experiment carried out in duplicate (n = 2 × 3).

### Biofilm formation and treatment of the biofilm

*S. aureus* Mu50 biofilms were formed as previously described^17^. In brief, overnight cultures in MH were centrifuged, the pellet was resuspended in double-concentrated MH (2 × MH) and diluted to an OD_590 nm_ of 0.2. Fifty microliter of the diluted bacterial suspension was transferred to the wells of a round-bottom 96-well microtiter plate (TPP). Control wells received 50 μl MilliQ. Wells used to evaluate pre-treatment received 50 μl of HAM solution (250 μM final concentration). Bacteria were allowed to adhere and grow without agitation for 4 h at 37 °C. After 4 h, medium was removed, and the adhered cells were washed with sterile physiological saline (0.9% NaCl; PS). After this washing step, control wells were filled with 50 μl 2 × MH and 50 μl MilliQ. Other wells were filled with 50 μl 2 × MH and 50 μl of HAM, and the plate was incubated for 20 h at 37 °C. To evaluate the effect of co-treatment on mature biofilms, control biofilms were formed in the absence of HAM, as described above. After 24 h of biofilm formation, the medium was removed and the wells were rinsed with PS. Control wells were either filled with 100 μl PS (untreated controls) or with 50 μl PS and 50 μl antibiotic solution. Wells used to evaluate the effect of pre-treatment were also filled with 50 μl PS and 50 μl antibiotic solution while wells used to evaluate combination treatment were filled with 50 μl of a HAM solution (250 μM final concentration) and 50 μl antibiotic solution. The plates were then incubated for an additional 24 h at 37 °C. After biofilm formation and treatment of the biofilms, the number of metabolically active cells were determined by resazurine staining (cell-titer blue, CTB)^49^ or by conventional plating^17^. To collect the cells for plating, plates were rinsed with PS, sessile cells were removed from the microtiter plate by two cycles of vortexing (5 min) and sonication (5 min) and the number of CFU/biofilm was determined by plating the resulting suspensions. The number of CFU/biofilm (for plating) or the fluorescence signal (for CTB staining) of the control biofilms was set to 100% and the results of the treated biofilms were compared to this. Each condition was tested in at least three wells in each assay, and each assay was carried out at least in triplicate (*n* ≥ 9).

### RNA-sequencing and data-analysis

Gene expression was measured in biofilms receiving no treatment, a treatment with VAN or HAM alone or a combination of VAN and HAM. For each condition 3 independent samples were incubated. RNA was extracted and approximately 30 ng of rRNA depleted RNA was used to create barcoded strand specific libraries with the Truseq stranded library preparation kit (Illumina, San Diego, USA). The libraries were equimolarly pooled and sequenced using an Illumina HiSeq 2000, generating 100 bp unpaired reads. After sequencing, the data were demultiplexed using sample specific nucleotide sequence thereby generating .fastq-files. The fastq-files were deposited in arrayexpress database (http://www.ebi.ac.uk/arrayexpress/) and are under the accession number: E-MTAB-3816.

After initial quality control, at least 10,000,000 high quality filtered reads were included in the analysis. Reads for each condition were mapped to the *S. aureus* Mu50 genome^50^. In order to be included in the analysis, reads must map to the entire gene with 100% similarity. In addition to these stringent mapping conditions, no mapping to the flanking regions is allowed. The number of reads assigned to a transcript were divided by the transcript length and normalized to the number of mapped reads to obtain reads per kb per million (RPKM) expression values.

Statistical analyses were performed using Baggerley’s t-test in the CLC genomics workbench software^51^. Statistical significance was defined as a p value smaller than 0.05. Only genes that were significantly differentially regulated (p < 0.05) and whose regulation differed at least 1.5 fold compared to the control were considered.

### Effect of HAM on membrane integrity

Membrane integrity was measured using propidium iodide as described^37^,^52^. *S. aureus* Mu50 was cultured overnight (16 h) in MH, centrifuged and the pellet was resuspended in PBS. The suspension was diluted to an OD_600_nm of 0.4. One ml aliquots received no treatment or were exposed to HAM and were placed at 37 °C for 10 min or 24 h. Heat killed cells (90 °C for 10 min) served as positive control. After 10 min or 24 h of treatment, propidium iodide was added for an additional 15 min to the samples and membrane damage was determined by measuring bacterial fluorescence (ex:485 nm; em:635 nm) by Envision microtiter plate reader (Perkin Elmer).

### Effect of HAM on lysostaphin susceptibility

Biofilms were formed and treated as described above. Cells were collected, resuspended and standardized to OD_600nm_ of 0.5. Lysostaphin (10 μg/ml) was added to the suspension and lysis was measured as a decrease in OD_620nm_ during incubation at 37 °C using an Envision microtiter plate reader (Perkin Elmer).

### Electron microscopy

Biofilms of *S. aureus* Mu50 strains were formed and treated as described above. After biofilm formation and treatment, biofilm cells were collected, washed and examined by transmission electron microscopy as previously described^53^. Morphometric evaluation of cell wall thickness was performed by using photographs of images obtained at a final magnification of 330,000. The thickness of the cell wall of at least 100 cells, in each condition, with nearly equatorially cut surfaces were measured using ImageJ software (http://imagej.nih.gov/ij/).

### Quantification of eDNA in the biofilm matrix

eDNA was quantified as previously described^54^, with minor modifications. In brief, biofilms were formed in the absence and presence of HAM as described above. Biofilm cells were washed and collected in Eppendorf protein LoBind microcentrifuge tubes (1.5 mL)

(Eppendorf AG, Hamburg, Germany). The biofilm cells were separated from the matrix by centrifugation at 5000 g for 10 min at 4 °C. The supernatant was aspirated and filtered through a 0.2 μm cellulose acetate filter (Whatman GmbH, Dassel, Germany) and the amount of eDNA was quantified using the Quantifluor dsDNA System kit (Promega, Madison, WI, USA). eDNA concentrations were normalized to the number of biofilm cells, determined by plate counting after 24 h of biofilm growth.

### Effect of DNAse on vancomycin susceptibility

Biofilms were formed in the absence and presence of HAM as described above. After 24 h of biofilm formation, biofilms were washed and a DNase solution (100 μg/ml) was added for 30 min. After this, the DNase solution was removed, cells were washed, a treatment with VAN was added and the cells were incubated for 24 h at 37 °C. After 24 h, treatment was removed, the biofilm cells were washed and the number of metabolically active cells was quantified using CTB staining^49^.

### Effect of exposure to VAN alone or in combination with HAM on hemolytic activity of *S. aureus*

Biofilm were formed and treated with HAM, VAN or a combination of VAN and HAM as described above. After 24 h of treatment, biofilms were washed with PBS, cells were collected and standardized to OD_600nm_ of 0.4. Two-hundred μl of each sample was incubated with 800 μL of 4% rabbit blood (Biotrading, Mijdrecht, The Netherlands). Samples treated with 10% SDS or PBS served as controls. All samples were incubated for 24 h at 37 °C. After incubation, samples were centrifuged (2 min at 1000 rpm), 100 μL of the supernatant was spotted on a microtiter plate and the OD_420nm_ was measured using the Envision microtiter plate reader (Perkin Elmer).

### *C. elegans* infection assay

*C. elegans* N2 (*glp-4*; *sek-1*) was propagated under standard conditions, synchronized by hypochlorite bleaching, and cultured on nematode growth medium using *E. coli* OP50 as food source as described previously^17^. The *C. elegans* assay was carried out as followed. Synchronized worms (L4 stage) were suspended in a medium containing 95% M9 buffer (3 g of KH_2_PO_4_, 6 g of Na_2_HPO_4_, 5 g of NaCl, and 1 ml of 1 M MgSO_4_ 7H_2_O in 1 liter of water), 5% brain heart infusion broth (Oxoid), and 10 μg of cholesterol (Sigma-Aldrich) per ml. Twenty-five μl of this suspension of nematodes (containing at least 25 nematodes) was transferred to the wells of a 96-well flat-bottomed microtiter plate. An overnight bacterial culture was centrifuged, resuspended in the assay medium, and standardized to 10^9^ CFU/ml. Next, 25 μl of this standardized suspension was added to each well, while 25 μl of sterile medium was added to the positive control. HAM (250 μM final concentration), VAN (20 μg/ml final concentration) or a combination of both was added to the test wells. The assay plates were incubated at 25 °C for up to 24 h. After 24 h, nematodes were collected and washed three times with M9 buffer supplemented with 1 mM sodium azide to prevent expulsion of the intestinal load and to remove surface-attached bacteria. The number of nematodes was then determined microscopically and nematodes were lysed in phosphate-buffered saline containing 400 mg 1.0 mm silicon carbide beads (VWR, Leuven, Belgium) and mechanically disrupted using a vortex shaker. Subsequently, lysates were serially diluted, plated on trypton soy agar supplemented with 7.5% NaCl and incubated at 37 °C. After 24 h, the number of CFU/nematode was determined. Each treatment was evaluated at least six times.

### Ethics Statement

All experiments were approved by the committee on the ethics of animal experiments of Faculty of Veterinary Medicine, Ghent University (Permit Number: EC2013/166). The animal experiments considered by the institutional ethical committee are according to specific Belgian (C-2013/24221) and European legislation (Directive 2010/63/EU of the European Parliament and of the Council on the protection of animals used for scientific purposes).

### *S. aureus* murine intramammary mastitis infection model

The *in vivo* effect of treatment with HAM alone or a combined treatment with HAM and CFL was evaluated using a murine intramammary *S. aureus* infection model, i.e. a model of *S. aureus* mastitis infection^33^. In brief, CD-1 dams (Harlan Laboratories Inc., Netherlands) were utilized 12–14 days after birth of the offspring. All inoculations were performed two hours post weaning under anesthesia. A mixture of oxygen and isoflurane (2–3%) was used for inhalational anesthesia of the lactating mice combined with a long-acting analgesic buprenorphine (10 μg/kg Vetergesic, Patheon UK Ltd, Swindon, UK). A syringe with 32-gauge blunt needle (Thiebaud Biomedical Devices, France) was applied to inoculate both L4 (on the left) and R4 (on the right) glands of the fourth abdominal mammary gland pair with approximately 150 CFU of *S. aureus* Newbould 305. Each orifice was exposed by a small cut at the near end of the teat and 100 μl of the inoculum was injected slowly through the teat canal. CFL was used as a preferred antibiotic in the field to treat mastitis infections in cattle^33^. In addition, a concentration of 100 μg/gland CFL was chosen since this concentration gave rise to a reduction in CFU/gland without full eradication. This allows observing additional killing for the combination, if present. The formulations containing HAM, CFL or a combination of CFL and HAM were instilled into the mammary gland of anesthetized mice using the desired dose (μg/gland) at 4 h after bacterial inoculation. All groups were composed of 5 mice (10 mammary glands). All mice were first sedated by administering a mixture of ketamine (100 mg/kg Anesketin, Eurovet Animal Health BV, Bladel, The Netherlands) with xylazine (10 mg/kg; Xylazini Hydrochloridum, Val d’Hony-Verdifarm, Beringen, Belgium) intraperitoneally and subsequently euthanized by cervical dislocation at 14 h post-treatment. The mammary glands (two per mouse) were harvested, weighed and homogenized on ice in sterile PBS using a tissue ruptor (QIAGEN Benelux BV, Netherlands). The mammary glands, which are physiologically separated, were considered as individual samples. Bacterial CFU counts were obtained by standard plating on trypton soy agar supplemented with 7.5% NaCl. Histological examination was conducted as previously described^55^,^56^.

### Statistical evaluation

The normal distribution of the data was checked by using the Shapiro-Wilk test. Normally distributed data were analyzed using a one-way ANOVA. Non-normally distributed data were analyzed using the Kruskal-Wallis test. Statistics were determined using SPSS software, version 22.0.

## Additional Information

**How to cite this article**: Brackman, G. *et al.* The Quorum Sensing Inhibitor Hamamelitannin Increases Antibiotic Susceptibility of *Staphylococcus aureus* Biofilms by Affecting Peptidoglycan Biosynthesis and eDNA Release. *Sci. Rep.* **6**, 20321; doi: 10.1038/srep20321 (2016).

## Supplementary Material

Supplementary Information
