# Supplementary material for: The Quorum Sensing Inhibitor Hamamelitannin Increases Antibiotic Susceptibility of Staphylococcus aureus Biofilms by Affecting Peptidoglycan Biosynthesis and eDNA Release
Source: Sci Rep. 2016 Feb 1;6:20321. doi: 10.1038/srep20321 (PMC4734334; doi:10.1038/srep20321)
Supplement: Supplementary Information [file srep20321-s1.pdf]

The Quorum Sensing Inhibitor Hamamelitannin Increases Antibiotic Susceptibility of *Staphylococcus aureus* Biofilms by Affecting Peptidoglycan Biosynthesis and eDNA Release

Gilles Brackman[a*], Koen Breyne[b], Riet De Rycke[c,d], Arno Vermote[e], Filip Van Nieuwerburgh[f], Evelyne Meyer[b], Serge Van Calenbergh[e], Tom Coenye[a]

Supporting information

Fig. S1: Effect of HAM on biofilm susceptibility of *S. aureus* JE2 and Newbould 305 against different types of antibiotics. The percentage CFU/biofilm ± s.d. (compared to untreated control biofilm) for *S. aureus* JE2 (black bars) or Newbould 305 (grey bars) biofilms exposed to vancomycin (VAN), cefazolin (CZ), cefalonium (CL), cephalexin (CFL), cefoxitin (Cfx), daptomycin (DAP), linezolid (LNZ), tigecycline (TGC), tobramycin (TOB) or fusidic acid (FA) alone or in combination with HAM. *: significantly increased killing was observed when biofilms were treated with the combination of the antibiotic and HAM compared to treatment with the antibiotic alone (n≥3; p <0.05).

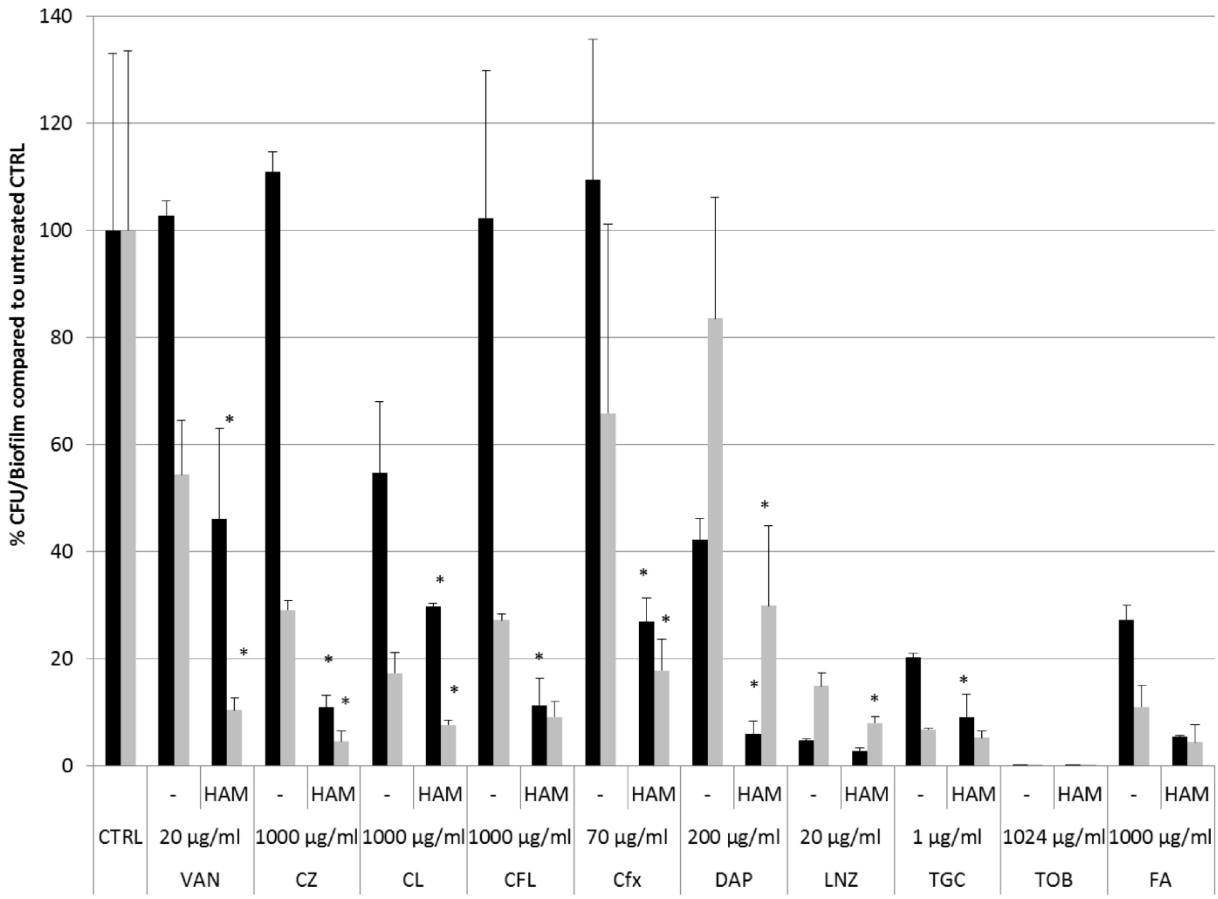

Fig. S2: Effect of HAM on growth. a) Effect of different concentrations of HAM on growth of *S. aureus* JE2 WT and mutant strains. b) Growth curve of *S. aureus* JE2 in the absence (black symbols) and presence of HAM (grey symbols). c) Cell-viability (quantified by CTB staining) of *S. aureus* JE2 WT and mutant strains exposed to HAM. Signals are presented as percentages (average ± s.d.) compared to the signal of an untreated biofilm. d) Cell-viability (quantified by CTB staining) of *S. aureus* JE2 WT and mutant strains exposed to VAN alone (black bars) or a combination of VAN and HAM (grey bars). Signals are presented as percentages (average ± s.d.) compared to the signal of an untreated biofilm. Exposure to a combination of VAN and HAM yielded significantly ($p < 0.05$) lower signal compared to exposure to VAN alone in the WT strain and in all mutant strains.

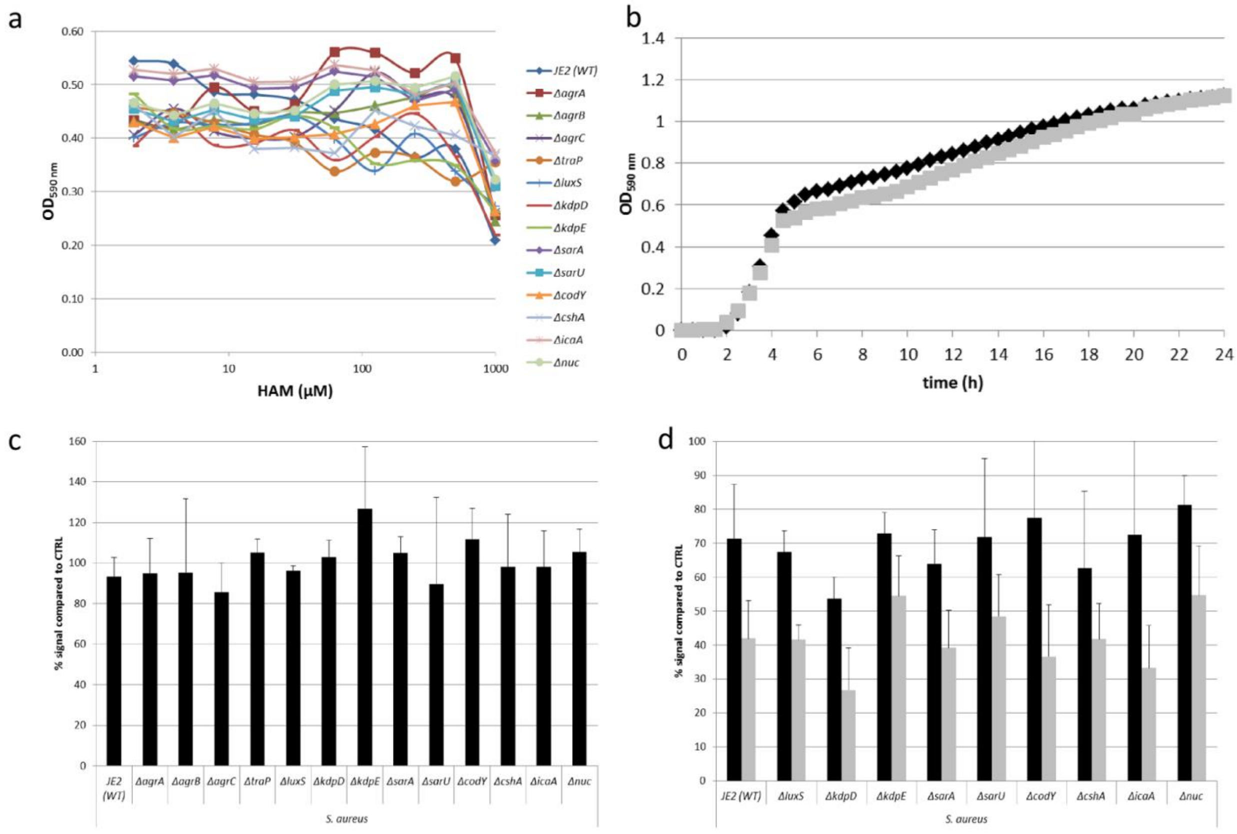

Fig. S3: Effect of HAM on biofilm susceptibility of strains belonging to different *Staphylococcus* species. Biofilms were exposed to HAM or VAN alone or a combination of VAN and HAM (COMB). Cell viability was quantified by CTB staining and signals are presented as percentages (average ± s.d.) compared to the signal of an untreated biofilm. No significant difference in fluorescence values were observed between treatment with VAN alone or in combination with HAM (p >0.01).

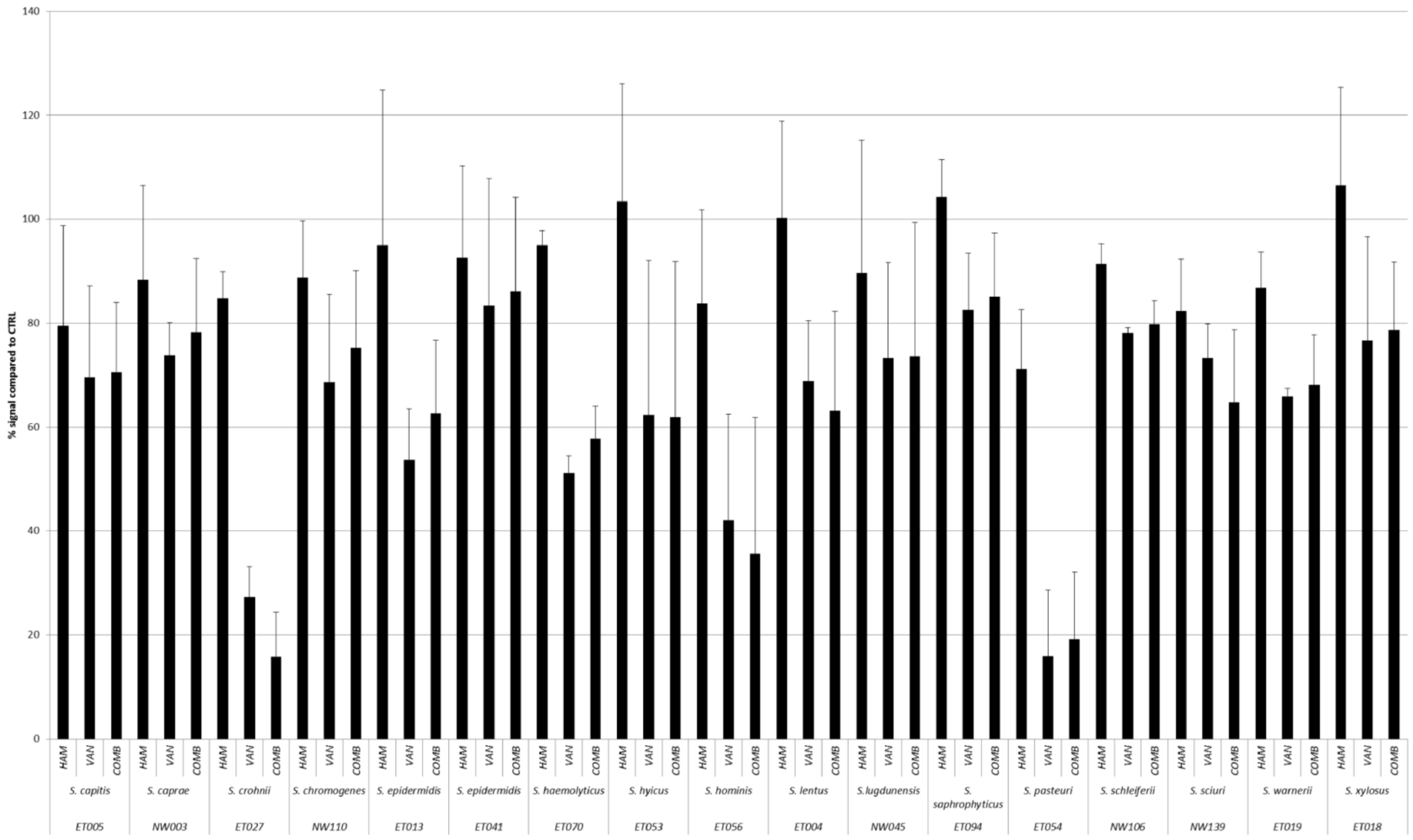

Fig. S4: Effect of HAM on biofilm susceptibility of *Burkholderia cenocepacia* LMG16656 and *Pseudomonas aeruginosa* PA01. Biofilms received no treatment (CTRL) or a treatment with HAM or tobramycine (TOB) alone or a combination of HAM and TOB (COMB) and the number of colony forming units /biofilm (CFU/BF) (average ± s.d.) was determined by plating. No differences in CFU/BF were observed between treatment with TOB alone or in combination with HAM (p >0.01).

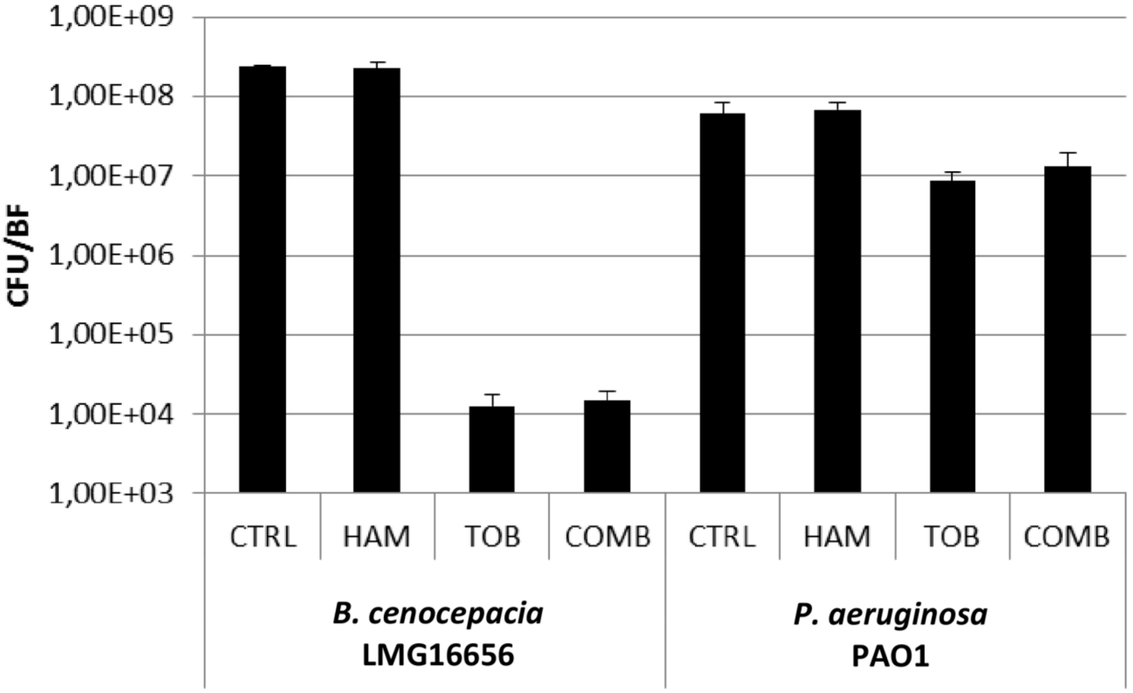

Fig. S5: Effect of HAM on growth (A) and membrane integrity (B) of *S. aureus* Mu50.

a) Growth curve of *S. aureus* Mu50 in the absence (black symbols) and presence of HAM (grey symbols).b) Membrane integrity was measured using propidium iodide (average ± s.d.) for *S. aureus* Mu50 receiving no treatment (CTRL) or a treatment with HAM or 0.1% SDS for 10 min or 24h. Membrane integrity of heat-treated (10 min, 90°C) *S. aureus* Mu50 cells was evaluated as a control. *: Propidium iodide fluorescence values differed significantly compared to the CTRL ($p < 0.01$). NS: no significant difference in propidium iodide values were observed compared to the CTRL ($p > 0.01$).

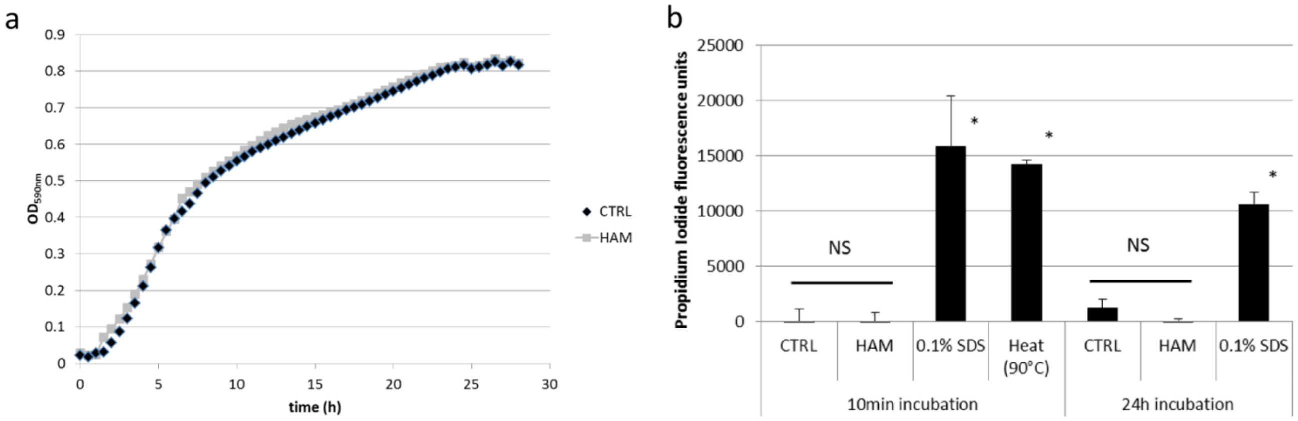

Fig S6: Pathway analysis for RNA sequence data of *S. aureus* Mu50 treated with VAN (A) or a combination of VAN and HAM (B) compared to the untreated strain. Green and red arrow indicate a upregulation and downregulation, respectively. The thickness of the lines is an indication of the fold change.

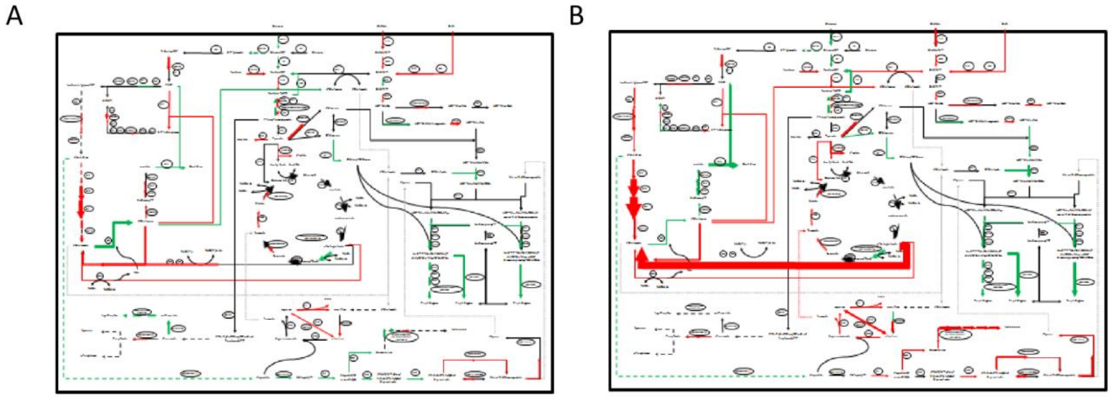

Fig. S7: Effect of treatment on survival of infected *C. elegans*. Percent survival (average ± s.d.) of uninfected (CTRL) and infected *C. elegans* receiving no treatment or a treatment with HAM, VAN or a combination of VAN and HAM (COMB). The results are expressed as the percent survival after 24 h of infection and treatment. *: treatment was significantly more effective than treatment with VAN alone ($P < 0.001$).

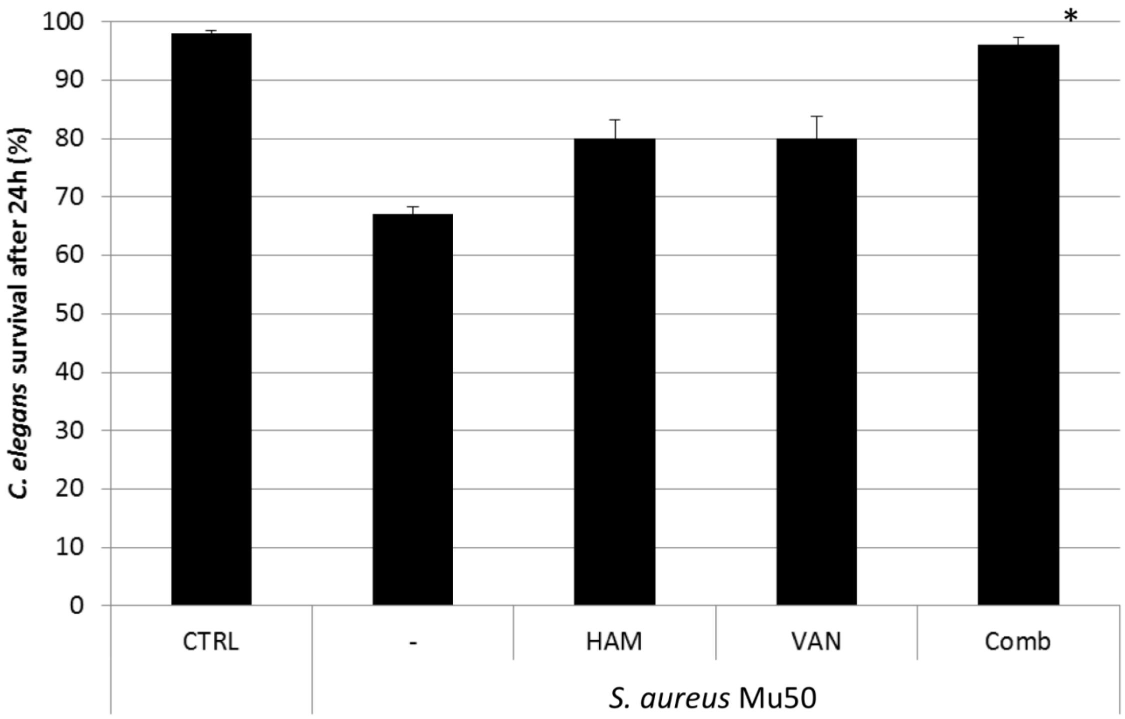

Fig. S8: Macroscopical histological evaluation. Macroscopical histological evaluation of mammary glands of mice infected with *S. aureus* Newbould 305 receiving no treatment or a treatment with HAM, CFL or a combination of CFL and HAM (COMB).

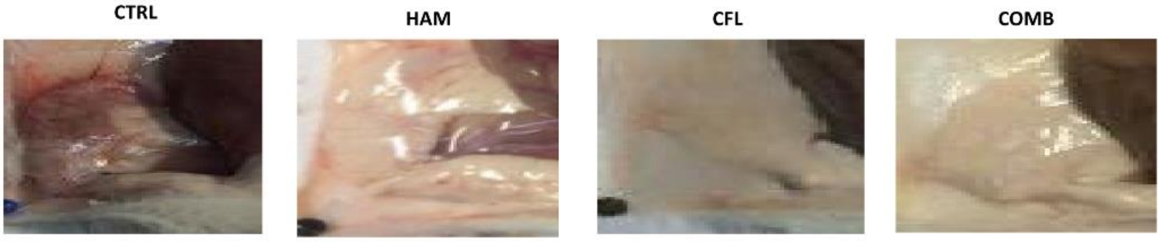

Table S1: MICs of different antibiotics (µg/ml) in the absence (-) and presence of HAM (250 µM) for *S. aureus* Mu50, JE2 and Newbould 305.

Antibiotics	*S. aureus*					
	Mu50		JE2		Newbould 305	
	-	HAM	-	HAM	-	HAM
VAN	2-4	2-4	2	2	1	1
CZ	32-64	32-64	1-2	1-2	0.5	0.5
CL	8	8	0.5	0.5	0.125	0.125
CFL	128	64-128	32	32	4	4
CFx	256	128-256	16	16	1	1
DAP	8	8	8	8	4	4
LNZ	2	0.5-1	2	1	2	2
TOB	256-512	128-256	0.5-1	1	1	1
FA	0.125	0.031-0.062	0.125	0.0625	0.125	0.125

Table S2: Genes that were differentially expressed compared to the CTRL after 2h treatment with HAM, VAN or a combination of both (COMB) as determined by RNA sequencing. The RNA sequence data are presented as the mean fold-change of 3 separate experiments. Data that met criteria for differentially-expressed genes (P value <0.05; >1.5-fold change) are included. NS (not significantly different) indicates no change compared to the control; red values indicate that genes are downregulated and green values indicate that genes are upregulated in these treatment conditions compared to the untreated control.

Feature ID	Experiment - Fold Change (normalized values)		
	HAM vs CTRL	VAN vs CTRL	COMB vs CTRL
aadD	-2,16	-2,42	-2,35
aapA	-1,77	-1,92	-2,42
accC	NS	NS	1,58
acpD	-3,52	-2,69	-4,05
acpP	-1,86	-2,02	-1,90
acsA	-1,95	-1,97	-2,05
acuA	-3,28	-2,61	-4,47
acuC	-1,90	-2,20	-2,81
adaB	-2,48	-2,38	-2,07
adhE	-1,74	-1,81	-2,28
agrA	1,56	NS	NS
agrC	1,69	1,63	NS
ahpC	NS	1,86	1,78
ahrC	-1,84	-1,60	-1,98
alaS	2,15	2,46	NS
ald	-2,74	-2,88	-3,04
aldA	NS	1,60	2,68
aldH	2,18	2,71	2,77
alsT	-1,84	-2,11	-2,05
ampA	-1,71	-1,57	NS
ansA	NS	NS	1,56
ant(9) (NC_002758 1763088..1763921)	NS	NS	-1,61
ant(9) (NC_002758 56794..57627)	NS	NS	-1,52
apt	2,21	1,94	NS
araB	NS	NS	1,79
arcA	NS	-1,63	-3,58
arcB	NS	-1,69	-3,16

arcC	NS	-1,51	-1,70
arcD	-1,76	-2,04	-3,20
argF	NS	NS	1,88
argG	NS	NS	-1,72
argH	NS	NS	-1,72
arlS	2,61	2,38	2,17
aroA	1,74	1,75	1,67
aroB	1,64	1,81	1,67
aroE	2,10	1,68	2,61
asd	NS	NS	-1,53
asp23	2,72	2,13	2,06
atpA	2,44	1,92	2,17
atpF	3,50	2,75	3,02
atpG	2,43	1,89	2,10
atpH	4,01	3,09	3,37
aur	3,92	3,51	5,20
betA	-2,24	-2,51	NS
bglA	-2,10	-2,28	-3,11
bleO	-1,58	-2,34	-2,01
bmfBB	2,51	2,13	1,95
braB	-1,70	-1,68	-2,17
bsaA	-1,72	-1,53	-1,64
butA	-2,09	-1,56	-4,40
capA	1,65	1,67	NS
capB	1,81	1,80	NS
capC	2,47	2,26	1,52
capE	NS	NS	-1,62
capF	NS	NS	-1,91
capI	1,54	NS	NS
capJ	NS	NS	-1,94
capK	NS	NS	-1,96
capL	NS	NS	-1,75
capO	NS	NS	-1,63
capP	NS	NS	-2,09
carB	NS	NS	2,35
cbf1	2,35	1,95	1,88
cbiO (NC_002758 2362853..2363713)	-2,03	-1,85	-2,41
ccpA	1,68	1,97	1,80
ccrA	-2,67	-2,64	-2,52
cdsA	NS	NS	2,14
cinA	3,14	4,12	4,49
clfB	1,50	1,66	1,92
clpC	3,93	2,55	2,48
clpL	1,57	1,99	NS
clpQ	1,67	1,64	2,12

clpX	2,53	2,55	3,17
coa	-1,99	-2,25	NS
codY	1,68	NS	1,75
comEB	NS	NS	-1,62
copA	NS	1,79	1,82
crtM	-1,63	-1,63	NS
crtN	-2,02	-2,35	-2,15
csbD	2,27	1,83	3,06
cspA	-1,93	-2,06	-2,72
cspC	NS	NS	-1,79
ctaB	-1,87	-2,13	-2,25
ctpA	4,20	5,43	9,80
ctsR	2,34	1,97	1,91
cudT	-1,81	NS	NS
cysK	NS	NS	1,66
cysM	NS	NS	4,29
cysS (NC_002758 595344..597222)	2,59	2,45	2,53
dapA	NS	1,78	NS
dapB	NS	NS	-1,55
dapD	-1,50	NS	-3,06
ddh	-1,56	-1,61	NS
ddl	1,74	1,93	2,11
def	NS	NS	1,96
deoD	-1,65	-1,63	-1,59
dhoM	NS	1,50	NS
dinG	3,07	2,84	2,98
div1b	4,22	4,10	5,63
dltA	NS	NS	1,67
dltB	1,96	1,81	2,69
dltC	2,58	2,26	3,28
dltD	2,91	2,55	3,49
dnaA	2,61	2,13	1,84
dnaB	1,59	1,54	NS
dnaE	1,77	NS	NS
dnaI	1,74	NS	NS
dnaJ	2,97	2,24	2,89
dnaK	6,96	5,37	4,30
dnaN	1,67	NS	1,52
dnlJ	3,01	3,06	4,28
dps	2,29	2,75	2,17
dra	-1,98	-1,80	-2,15
drm	-1,89	-2,07	-2,03
drp35	-1,58	NS	2,03
ebpS	3,79	3,86	4,29
engB	4,52	4,29	4,59

fabD	NS	NS	1,72
fabH	NS	NS	1,92
fabI	-1,51	NS	NS
fbaA	-1,80	-1,84	-2,11
fbp	1,76	1,99	2,78
fdh	NS	-1,79	NS
femA	2,58	2,78	3,42
femB	2,09	1,86	3,31
feoB	NS	-2,49	-2,83
fer	-2,21	-1,59	NS
ffh	2,63	2,68	4,88
fhuA	-2,14	-2,67	-3,53
fhuB	-1,81	-2,17	-2,69
fhuG	-1,73	-2,15	-2,36
fmhA	NS	1,66	NS
fmhB	2,55	2,61	3,25
fmt	1,82	2,20	4,01
fmtC	-1,87	-1,79	NS
fnb (NC_002758 2642289..2645456)	NS	NS	1,94
fnbB	NS	NS	1,78
fni	NS	NS	-1,83
folB	-1,67	-1,93	NS
folC	3,03	2,29	2,67
folD	NS	1,51	1,55
folK	-1,85	-1,77	-1,74
folP	-1,84	-2,15	NS
fosB	NS	1,95	NS
frr	4,01	3,92	5,76
fruA	-1,63	-1,78	-1,86
ftsA	2,91	2,53	2,96
ftsH	2,58	2,44	2,96
ftsL	1,71	1,80	1,84
ftsZ	1,90	1,80	1,81
fumC	-1,61	-1,64	-1,87
gap	-1,61	NS	-1,55
gapR	-1,81	NS	NS
gatA	1,75	1,57	2,43
gatB	2,48	2,02	3,63
gatC (NC_002758 2042756..2043109)	NS	1,60	2,14
gbsA	-2,64	-2,93	NS
gcvT	3,00	3,32	10,30
gerCC	2,28	1,89	2,34
gid	2,22	2,14	3,79
gidA	2,46	2,16	3,20
gidB	3,14	2,74	3,85

glcA	-1,98	-2,05	-1,78
glcT	1,91	2,48	1,98
glmS	NS	NS	-1,54
glmU	NS	-1,92	-1,58
glnA	1,65	4,13	1,78
glnR	5,16	7,08	2,99
glpD	-1,78	-1,67	NS
glpF	-3,62	-3,80	-4,49
glpK	-1,78	-2,01	NS
glpP	4,91	4,83	7,71
glpQ	1,54	1,55	NS
glpT (NC_002758 382413..383822)	-2,39	-2,39	-2,83
gltB	NS	-2,65	-15,33
gltC	NS	NS	-1,71
gltD	-3,39	-4,49	-26,12
gltT	-2,20	-2,61	-3,11
gltX	1,74	1,63	1,67
glvC	-1,73	NS	NS
glyS	NS	-1,86	-3,37
gmk	NS	NS	-1,52
gntK	-3,81	-4,44	-5,04
gntP	-1,73	-2,38	-2,48
gntR	-4,85	-4,19	-5,33
gpmA	-2,01	-1,85	-2,29
greA	2,27	2,18	2,44
groEL	NS	-1,91	NS
groES	NS	NS	1,67
grpE	5,95	4,49	5,03
gtaB	-2,01	-2,10	-2,86
guaA	NS	NS	-1,64
gudB	-1,52	-1,65	-1,56
gyrA	NS	NS	-1,65
hemC	2,03	1,79	1,55
hemD	2,14	1,89	1,73
hemH	1,79	1,76	2,44
hemL	-1,60	-1,72	-1,65
hemN	1,72	1,55	2,09
hemX	1,88	1,85	NS
hisB	NS	NS	-1,91
hisH	NS	NS	-2,01
hisI	2,32	1,89	NS
hisZ	NS	NS	-1,92
hit	NS	1,62	NS
hld	NS	NS	1,73
hlgA	NS	4,09	NS

hlgB	NS	4,49	NS
hlgC	NS	5,96	2,11
hmrA	-1,87	-1,69	-1,73
holA	3,53	2,85	3,39
hprK	-1,80	-1,70	NS
hrcA	2,76	2,36	2,27
hsdM	-1,61	-1,67	-2,06
hsdR	NS	NS	-1,80
hsdS	3,41	2,77	2,19
hslU	3,68	3,35	4,34
htrA	-1,70	-1,52	NS
htsA	1,61	NS	NS
htsB	NS	-1,89	-2,37
htsC	NS	-2,55	-2,54
hutH	-1,86	-2,00	-2,36
hutI	-4,04	-5,64	-12,19
hutU	-3,67	-4,70	-9,53
icaR	2,49	2,69	2,77
ileS	NS	NS	1,63
ilvA	NS	-1,59	-4,42
ilvB	NS	NS	-2,40
ilvC	NS	NS	-3,38
ilvD	NS	NS	-2,82
infA	1,78	1,53	1,86
infC	3,43	2,87	4,93
int (NC_002758 2125047..2126135)	1,96	1,81	2,85
int (NC_002758 436045..437289)	2,69	2,42	-1,98
int (NC_002758 917481..918581)	-1,51	-1,71	-1,53
ipk	NS	NS	-1,66
isaA	-1,63	NS	NS
isaB	2,68	2,42	2,75
isdI	NS	-1,86	-2,66
ispA	2,28	2,03	2,53
ispD (NC_002758 292852..293619)	1,78	1,72	1,78
kdpA	-3,38	-3,42	-6,60
kdpB	NS	-1,59	-1,95
kdpB(SCCmec)	NS	-1,64	-3,21
kdpC	-2,72	-2,85	-3,12
kdpC(SCCmec)	NS	-1,57	-2,41
kdpE (NC_002758 2209741..2210487)	2,70	2,63	2,15
kdpE (NC_002758 73444..74190)	-2,09	-2,02	-1,68
ksgA	1,97	1,81	1,98
lacA	NS	3,74	1,80
lacB	NS	3,18	1,59
lacC	2,31	4,76	1,81

lacD	2,45	5,08	2,09
lacE	1,76	3,63	NS
lacF	2,69	4,92	2,24
lacG	NS	2,50	-1,78
lctE	NS	-1,53	-2,32
lctP	-2,67	-3,05	-3,37
leuA	NS	NS	-4,10
leuB	NS	NS	-4,05
leuC	NS	NS	-3,93
leuD	NS	NS	-3,80
lexA	NS	NS	-1,66
lip	2,77	2,83	1,62
lipA	-1,53	-1,59	NS
llm	-1,53	-1,55	NS
lpl9	2,56	2,29	2,96
lrgA	-3,15	-3,09	NS
lrgB	-2,30	-2,44	NS
lspA	-1,63	-1,75	NS
lysC	NS	1,89	NS
lytH	2,19	2,21	1,85
lytM	NS	2,01	3,48
lytS	-2,11	-1,85	-2,72
malA	NS	-1,55	-1,68
malR	-2,48	-2,13	-2,81
map	-2,38	-2,30	-2,36
mecA	3,81	3,46	3,42
mecI	5,85	4,14	4,36
menB	-1,71	-1,72	NS
menC	-1,57	NS	-1,65
menD	NS	1,54	2,27
menE	-2,49	-2,41	-2,19
metE	1,58	1,57	NS
metK	-1,53	-1,67	NS
metS	NS	NS	-1,54
mfd	1,55	NS	1,67
miaA	1,74	1,82	2,02
mnhA	-1,71	-1,75	-1,93
mnhB	-1,66	-2,02	-1,84
mnhC	-1,68	-1,93	-1,94
mnhD	-1,58	-1,88	-1,87
mnhE	NS	-1,63	-1,78
mnhF	NS	-1,72	-1,71
mnhG	NS	-1,53	-1,67
moaB	NS	-1,53	-1,55
moaC	-2,87	-2,69	-3,78

modA	1,71	1,68	1,57
modC	1,82	1,67	1,66
msmX	-1,84	NS	NS
msrA	1,58	2,10	1,67
msrR	NS	1,80	3,57
mtlA	NS	-2,23	-1,92
mtlD	-1,65	-2,51	-2,13
mtlF	-2,33	-3,97	-4,66
murB	-2,07	-2,25	-1,88
murD	1,85	1,75	2,39
murE	-1,67	NS	NS
murF	1,72	1,55	1,96
murG	2,31	2,29	2,43
murQ	NS	-1,69	NS
mutL	4,99	5,06	8,11
mutS	3,18	3,42	4,88
mutS2	4,77	3,72	5,25
mvaA	-1,91	-2,10	-1,82
mvaD	NS	NS	3,40
mvaK1	-1,70	NS	2,05
mvaK2	1,52	1,61	4,56
mvaS	-1,87	-1,95	NS
nadE	2,65	2,59	3,34
nagA	-2,65	-2,47	-1,88
nagB	-1,54	-1,61	-1,51
nanA	-2,27	-2,95	-1,87
narG	NS	-2,41	NS
narH	NS	-2,31	NS
narI	-2,67	-2,69	-2,86
narK	-3,28	-2,88	-2,81
narQ	-1,81	NS	-1,54
nasF	-2,30	-2,19	-2,16
ndhF	-1,67	-2,19	-2,45
ndk	-3,75	-3,56	-3,93
norA	-2,50	-2,29	-2,03
nrdE	2,33	2,00	NS
nrdF	2,15	2,04	1,66
nrdI	NS	NS	-1,56
nrdR	NS	NS	-1,68
nrgA	-1,73	NS	-2,37
nth	3,26	3,07	4,37
nuc (NC_002758 894238..894975)	NS	NS	1,61
nupC	-2,92	-3,29	-4,54
nusB	4,12	4,50	4,53
nusG	2,27	1,93	2,20

obgE	-1,95	-2,39	-2,50
odhB	5,11	4,37	4,19
oppB (NC_002758 1034274..1035251)	-3,22	-3,50	-5,81
oppB (NC_002758 1043913..1044926)	-1,61	NS	-1,65
oppD	NS	NS	-1,63
oppF (NC_002758 1037358..1038350)	1,73	NS	-1,61
opuCA	-1,69	-1,73	-1,94
opuCB	NS	-1,56	NS
opuCC	-1,71	-2,01	-2,12
opuCD	-2,13	-2,49	-2,68
opuD	-2,15	-2,26	NS
panB	-2,38	-1,52	-2,39
parC	2,96	2,33	3,36
pbp2	2,78	3,54	5,56
pbp3	2,29	1,84	2,24
pbp4	1,58	1,82	2,62
pbpA	2,56	2,28	2,94
pbuX	NS	2,14	NS
pckA	1,75	1,88	2,24
pcp	-3,60	-3,06	-4,13
pcrA	3,91	4,06	5,65
pcrB	NS	1,77	2,24
pdp	NS	-1,51	NS
pfkA	-1,66	-1,57	NS
pflA	NS	1,86	NS
pflB	NS	1,65	NS
pgi	1,90	2,16	2,14
phoR	2,23	2,12	2,70
plsX	1,72	1,63	2,33
polA	2,14	2,28	2,34
polC	1,66	1,83	2,13
potD	NS	-1,60	NS
ppnK	1,56	1,64	NS
pre	2,03	1,63	NS
priA	1,80	1,58	1,88
proS	NS	NS	1,82
prsA	5,32	7,85	8,78
pstB	NS	NS	3,12
ptaA	-1,91	-2,19	-2,31
pth	-1,51	NS	NS
ptsG	-2,28	-2,28	-2,03
purA	NS	NS	2,05
purB	NS	NS	2,05
purC	-1,98	-1,70	NS
purD	NS	NS	-1,53

purF	-1,86	-1,70	NS
purK	NS	NS	2,06
purL	-2,07	-1,99	NS
putP	-2,10	-2,32	-3,97
pycA	NS	NS	-1,73
pyrAA	NS	-1,71	2,09
pyrB	NS	NS	3,58
pyrC	NS	-1,84	2,29
pyrE	1,74	1,66	4,10
pyrF	1,85	1,70	4,71
pyrG	-1,55	-2,13	NS
pyrH	NS	NS	1,90
pyrP	-1,79	-2,43	2,11
pyrR	-3,34	-4,16	NS
qoxB	NS	-1,85	-2,08
qoxC	-1,82	-2,54	-2,64
queA	NS	-1,85	-1,53
rbfA	2,24	2,08	2,67
rbgA	1,75	2,23	2,26
recA	NS	NS	-1,79
recG	1,88	2,32	2,04
recN	2,13	2,01	1,66
recQ	4,69	3,67	4,12
recR	-1,50	-1,54	-1,73
recU	2,74	3,49	6,23
recX	2,70	3,20	4,26
relA	2,37	2,41	2,04
ribA	1,80	2,17	2,86
ribB	2,45	2,86	4,09
ribD	NS	1,81	2,50
ribH	NS	1,51	1,80
rluB	2,85	2,20	2,39
rnhB	1,85	2,07	2,46
rnpA	NS	NS	1,79
rot	-1,67	-1,78	-2,14
rplA	NS	NS	1,66
rplB	2,05	1,81	3,59
rplC	2,62	2,34	5,19
rplD	2,73	2,37	5,23
rplE	3,83	3,18	7,33
rplF	2,97	2,63	5,26
rplI	3,53	2,80	3,15
rplL	NS	NS	1,62
rplN	2,69	2,25	4,81
rplP	2,63	2,35	4,80

rplQ	2,27	1,96	2,02
rplR	2,46	2,23	4,30
rplS	-2,11	-2,77	NS
rplT	3,69	3,07	6,00
rplV	3,13	2,67	6,19
rplW	3,54	3,23	6,81
rplX	3,64	3,15	7,43
rpmB	NS	NS	-1,52
rpmC	4,01	3,32	7,47
rpmD	1,65	NS	2,45
rpmE2	-2,17	-2,77	-1,78
rpmG (NC_002758 1661950..1662150)	3,40	3,04	4,83
rpmH	-1,56	-1,68	NS
rpmI	3,88	3,06	5,73
rpmJ	2,08	1,90	2,05
rpoA	2,32	2,03	2,26
rpoC	1,92	1,50	1,72
rpsA	NS	NS	1,57
rpsB	1,78	1,80	5,06
rpsC	2,84	2,44	5,45
rpsE	1,89	1,70	3,03
rpsF	-1,60	-1,79	NS
rpsH	3,37	2,94	6,32
rpsJ	1,85	1,68	3,66
rpsK	2,13	1,97	2,29
rpsM	2,11	1,96	2,27
rpsN (NC_002758 1410613..1410933)	2,71	2,28	2,92
rpsN (NC_002758 2371472..2371708)	2,74	2,52	5,49
rpsO	NS	-1,50	1,53
rpsP	1,63	1,74	3,03
rpsQ	4,81	3,91	8,82
rpsS	2,83	2,40	5,73
rpsT	2,61	2,55	3,90
rsbU	NS	1,55	NS
rsbW	1,51	NS	NS
ruvB	NS	-1,73	-1,51
saeR	2,71	2,73	1,68
saeS	4,04	4,18	2,37
sak	-1,59	-1,63	-1,57
sarA	3,89	4,03	4,35
sarH1	2,90	2,68	2,37
sarR	4,80	3,97	2,02
SAV0003	NS	NS	-1,80
SAV0007	-2,25	-1,73	-2,63
SAV0010	-2,45	-2,19	-4,04

SAV0011	NS	NS	-2,36
SAV0012	-1,75	-1,68	-2,31
SAV0013	NS	NS	-1,74
SAV0014	1,91	1,70	1,69
SAV0020	3,01	2,43	2,82
SAV0021	3,08	2,59	2,81
SAV0022	-3,09	-2,55	-2,33
SAV0024	NS	1,60	NS
SAV0025	2,85	2,51	2,22
SAV0026	-2,02	-2,03	-2,22
SAV0032	-1,70	-1,69	NS
SAV0033	-2,05	-2,69	-2,70
SAV0038	-1,62	NS	NS
SAV0039	NS	NS	2,15
SAV0040	2,46	2,22	3,51
SAV0049	-1,81	-1,75	-1,98
SAV0050	-1,77	-1,84	-1,95
SAV0051	NS	NS	1,52
SAV0058	-2,24	-2,39	-2,18
SAV0063	-1,74	-1,93	NS
SAV0064	-1,96	-1,89	NS
SAV0065	3,25	3,37	2,90
SAV0069	-1,80	-1,89	-1,96
SAV0075	NS	NS	-1,67
SAV0076	-1,75	-1,89	-1,78
SAV0077	-1,88	-1,95	-1,69
SAV0079	NS	-1,54	NS
SAV0080	-2,16	-2,25	-2,14
SAV0082	3,78	4,02	4,28
SAV0088	-1,76	-2,06	-1,73
SAV0089	-2,59	-2,89	-2,92
SAV0093	2,59	2,31	1,77
SAV0094	1,83	1,54	NS
SAV0099	2,45	2,23	NS
SAV0100	2,22	1,88	NS
SAV0102	-2,01	-2,04	-1,80
SAV0103	-2,37	-2,50	-2,10
SAV0104	-1,99	-1,83	-1,75
SAV0105	-3,21	-2,65	-3,36
SAV0106	-1,96	-1,73	-1,96
SAV0109	-2,33	-2,73	-2,10
SAV0119	-2,29	-2,34	-2,31
SAV0122	-1,94	-2,21	-2,14
SAV0123	-2,26	-2,11	-2,39
SAV0124	-2,32	-2,44	-2,45

SAV0125	-3,23	-2,53	-3,07
SAV0128	-2,36	-2,40	-2,50
SAV0129	-2,29	-2,42	-3,04
SAV0132	-2,10	-2,33	-1,95
SAV0135	-2,68	-2,73	-3,11
SAV0137	NS	NS	-1,54
SAV0140	-1,66	-1,86	-1,59
SAV0141	NS	-1,81	NS
SAV0145	-2,86	-3,09	-3,76
SAV0146	NS	1,75	NS
SAV0147	2,25	2,35	2,07
SAV0166	-1,65	-2,19	-3,84
SAV0169	NS	-1,58	-1,54
SAV0170	-1,73	-1,57	NS
SAV0171	1,84	2,05	5,97
SAV0172	NS	NS	2,53
SAV0174	NS	NS	1,96
SAV0176	-2,82	-2,94	-3,55
SAV0178	-1,76	-1,53	NS
SAV0179	NS	NS	-1,66
SAV0180	-1,52	NS	-1,90
SAV0181	-1,93	-1,98	-2,40
SAV0185	NS	NS	-1,82
SAV0186	-3,29	-3,42	-5,18
SAV0187	NS	NS	2,87
SAV0188	NS	1,85	4,73
SAV0190	-3,29	-3,26	-2,60
SAV0192	-2,22	-2,43	-1,72
SAV0193	-2,48	-2,69	-2,12
SAV0194	-2,02	-2,28	-2,16
SAV0200	-4,16	-4,26	-4,21
SAV0202	NS	3,91	4,61
SAV0203	2,34	2,90	3,56
SAV0204	7,90	9,04	7,30
SAV0209	NS	-1,84	NS
SAV0210	1,77	1,84	NS
SAV0212	NS	2,35	NS
SAV0215	-1,70	-1,85	-1,72
SAV0216	-1,81	-2,07	-2,11
SAV0217	-1,71	-1,81	-1,95
SAV0218	-1,73	-1,84	-1,83
SAV0219	-1,76	-1,99	-2,19
SAV0228	NS	1,61	NS
SAV0229	NS	NS	1,61
SAV0231	NS	-9,57	-18,17

SAV0232	NS	-6,11	-11,29
SAV0233	NS	-4,81	-9,21
SAV0234	NS	-3,84	-7,72
SAV0235	NS	-3,25	-7,96
SAV0236	-1,76	-2,01	-2,10
SAV0237	2,08	2,29	2,90
SAV0238	1,50	1,64	2,13
SAV0239	2,13	2,58	2,95
SAV0240	2,35	2,82	3,14
SAV0242	-2,57	-3,16	-3,72
SAV0243	-2,15	-2,12	NS
SAV0245	3,15	2,90	3,50
SAV0246	3,94	3,23	3,60
SAV0250	-1,51	-1,61	-1,78
SAV0252	1,63	NS	1,62
SAV0253	NS	-1,83	-1,55
SAV0254	NS	NS	2,23
SAV0256	1,68	1,53	1,73
SAV0257	2,36	2,05	2,26
SAV0258	2,25	1,98	2,06
SAV0264	-3,46	-3,35	-5,39
SAV0265	-1,74	NS	-2,30
SAV0267	-2,17	-2,32	-2,43
SAV0270	-1,80	-1,92	-1,70
SAV0271	-2,89	-2,96	-2,82
SAV0273	2,79	2,92	2,59
SAV0274	-2,37	-2,47	-1,99
SAV0275	NS	-1,93	-1,72
SAV0277	NS	NS	1,57
SAV0278	NS	NS	2,03
SAV0279	NS	NS	1,71
SAV0280	2,29	2,40	1,87
SAV0281	-1,67	NS	-2,19
SAV0282	-1,53	-2,30	-3,53
SAV0283	3,45	3,02	2,37
SAV0284	2,31	1,99	1,56
SAV0286	2,51	2,69	NS
SAV0287	2,09	1,95	NS
SAV0290	2,37	2,48	NS
SAV0291	3,48	3,93	2,72
SAV0292	2,75	2,87	NS
SAV0294	-1,57	-1,93	NS
SAV0295	2,61	2,38	4,52
SAV0297	-1,66	-1,63	-2,19
SAV0299	-1,93	NS	NS

SAV0303	3,46	3,09	2,47
SAV0305	NS	-1,81	NS
SAV0306	-1,84	-1,67	NS
SAV0308	-1,54	-1,78	-1,50
SAV0309	-1,66	-2,13	-1,96
SAV0310	-2,92	-2,91	-3,29
SAV0313	NS	-1,73	NS
SAV0314	-2,53	-3,74	-2,27
SAV0316	NS	-1,60	NS
SAV0318	-2,18	-2,59	-2,27
SAV0319	-5,59	-6,98	-18,94
SAV0322	-2,03	-2,06	-1,66
SAV0323	-2,05	-2,55	-1,57
SAV0324	NS	-1,78	NS
SAV0325	NS	-1,52	NS
SAV0326	NS	-1,64	NS
SAV0330	NS	NS	-1,60
SAV0333	-2,10	-2,15	-2,33
SAV0334	-1,55	-1,89	-1,87
SAV0338	-2,11	-1,80	-1,93
SAV0339	-2,07	-2,14	-2,22
SAV0340	-2,50	-2,63	-2,67
SAV0341	-4,70	-3,71	-4,90
SAV0342	-2,86	-2,11	-5,71
SAV0343	2,72	3,56	NS
SAV0346	NS	1,73	1,68
SAV0347	NS	1,99	NS
SAV0348	NS	3,27	2,56
SAV0349	NS	-3,12	NS
SAV0352	-2,32	-2,49	-2,21
SAV0353	-2,25	-1,94	-3,07
SAV0354	-1,79	-1,82	NS
SAV0355	NS	NS	-1,55
SAV0358	NS	NS	-1,64
SAV0359	NS	NS	-1,77
SAV0361	1,54	1,68	3,16
SAV0363	-4,32	-4,40	-3,96
SAV0364	-2,67	-3,35	-6,02
SAV0370	2,46	3,62	1,84
SAV0373	NS	NS	-1,93
SAV0374	-4,50	-5,41	-5,66
SAV0376	-2,31	-2,53	-2,08
SAV0377	-2,88	-3,03	-2,96
SAV0378	2,89	2,85	2,42
SAV0383	-1,62	-1,80	NS

SAV0385	3,25	3,32	2,98
SAV0386	3,82	3,96	3,82
SAV0387	1,74	1,71	1,84
SAV0393	1,78	1,78	NS
SAV0394	-2,07	-2,13	-2,53
SAV0395	-2,21	-2,51	-2,71
SAV0396	NS	-2,89	-2,28
SAV0397	-2,07	-3,08	-3,99
SAV0402	-1,65	-1,88	-1,84
SAV0404	2,00	1,87	NS
SAV0406	NS	NS	-1,83
SAV0410	-1,69	-1,80	-1,72
SAV0413	2,68	2,31	2,03
SAV0414	2,37	2,04	1,70
SAV0416	2,07	1,79	1,69
SAV0418	3,17	2,47	2,73
SAV0419	1,91	1,63	NS
SAV0421	-1,60	NS	-1,66
SAV0434	2,00	2,13	2,29
SAV0435	NS	NS	-3,46
SAV0446	3,43	3,03	3,85
SAV0447	5,58	5,03	5,72
SAV0448	4,69	4,51	4,83
SAV0449	-3,57	-3,92	-3,87
SAV0450	-1,87	-1,98	-1,93
SAV0453	NS	-1,82	-1,61
SAV0454	-2,47	-2,72	-3,28
SAV0455	-3,98	-3,15	-5,27
SAV0456	-1,85	-2,15	-2,02
SAV0457	-1,67	-2,23	-1,95
SAV0458	NS	1,89	-2,41
SAV0462	-2,05	-1,69	NS
SAV0465	NS	-1,66	NS
SAV0466	-2,31	-2,26	-2,28
SAV0467	-1,93	-1,89	-2,09
SAV0468	-2,01	-2,11	-2,42
SAV0470	-2,25	-1,97	-2,21
SAV0475	NS	-1,62	NS
SAV0476	-2,13	-2,20	-1,90
SAV0477	-3,71	-3,37	-4,77
SAV0481	-1,87	-1,75	-1,94
SAV0483	-1,82	-2,20	-2,62
SAV0486	NS	NS	-1,63
SAV0487	-1,67	-1,88	-2,26
SAV0491	NS	-1,72	-1,87

SAV0497	-1,58	-2,14	-2,65
SAV0506	2,86	2,81	2,56
SAV0507	4,28	4,35	3,72
SAV0509	1,64	NS	NS
SAV0510	2,38	2,14	2,23
SAV0519	-1,58	-2,04	NS
SAV0520	NS	-1,81	NS
SAV0523	3,43	2,94	2,80
SAV0524	3,75	2,62	2,73
SAV0527	2,12	2,04	2,47
SAV0531	2,99	2,74	2,64
SAV0532	2,75	2,48	2,21
SAV0533	3,47	3,22	3,04
SAV0549	NS	1,65	NS
SAV0553	-1,84	-2,35	-3,28
SAV0558	-2,73	-2,10	-2,65
SAV0559	-1,65	NS	-1,52
SAV0560	2,53	2,80	2,86
SAV0564	2,79	2,90	2,48
SAV0572	-1,86	-2,43	-2,34
SAV0574	NS	NS	-8,78
SAV0578	-2,80	-2,35	-1,54
SAV0580	-1,90	-1,50	NS
SAV0584	-2,92	-3,13	-2,38
SAV0586	-1,66	NS	NS
SAV0593	2,42	2,62	6,86
SAV0595	NS	1,59	NS
SAV0596	1,74	1,82	1,63
SAV0597	3,14	3,07	3,00
SAV0598	3,98	3,92	3,42
SAV0599	3,49	3,06	3,00
SAV0600	-1,51	-1,56	NS
SAV0608	2,51	2,29	2,61
SAV0609	1,59	1,59	NS
SAV0610	-2,50	-2,32	-2,46
SAV0612	2,90	3,03	2,44
SAV0613	NS	1,84	NS
SAV0615	-2,75	-2,92	-3,28
SAV0617	-1,71	-1,73	NS
SAV0620	-2,11	NS	NS
SAV0623	-1,78	-1,63	NS
SAV0624	-1,82	-1,53	NS
SAV0625	-1,77	-1,76	NS
SAV0626	-2,18	-2,19	NS
SAV0627	-2,17	-1,82	-1,60

SAV0628	-1,99	-1,93	-1,80
SAV0631	4,69	3,13	1,57
SAV0632	NS	-1,52	-2,86
SAV0633	-1,79	-1,87	-3,85
SAV0635	-1,82	NS	NS
SAV0643	-1,59	-1,54	-1,74
SAV0644	-2,33	-2,30	-2,18
SAV0645	-2,37	-2,65	-3,63
SAV0646	-2,28	-2,30	-2,48
SAV0650	-1,54	-1,52	NS
SAV0653	-1,97	-1,79	-1,60
SAV0654	NS	-1,65	NS
SAV0655	1,78	1,82	2,66
SAV0656	2,01	2,12	1,84
SAV0657	-1,94	-1,75	-1,68
SAV0659	3,12	3,02	3,16
SAV0660	2,46	2,18	2,33
SAV0663	NS	1,51	1,54
SAV0667	3,06	3,01	2,96
SAV0669	-1,66	-1,78	-1,74
SAV0670	-1,54	-1,58	-1,58
SAV0671	2,62	3,28	2,05
SAV0672	2,25	2,24	1,75
SAV0673	NS	-1,68	NS
SAV0674	-2,00	-2,41	-1,82
SAV0677	1,75	1,71	NS
SAV0679	-1,58	-1,81	NS
SAV0680	-1,76	NS	-1,52
SAV0681	NS	NS	1,74
SAV0682	2,26	2,57	3,35
SAV0686	2,55	2,38	2,54
SAV0688	-1,84	-1,91	-1,77
SAV0690	-2,49	-2,68	-2,91
SAV0691	-1,91	-2,18	-2,54
SAV0696	2,77	3,39	3,62
SAV0697	-1,98	-1,79	-1,72
SAV0702	NS	NS	1,58
SAV0704	-1,70	-1,71	-1,59
SAV0707	1,96	2,25	NS
SAV0708	3,91	4,07	2,52
SAV0709	1,77	3,93	1,74
SAV0710	1,66	1,65	1,64
SAV0711	1,70	1,72	1,79
SAV0712	1,85	2,23	2,04
SAV0713	-2,37	-2,22	-2,55

SAV0714	1,51	NS	NS
SAV0715	3,69	3,44	2,77
SAV0717	1,74	1,63	NS
SAV0718	1,86	1,63	NS
SAV0719	4,94	4,11	6,39
SAV0720	1,86	1,74	1,69
SAV0723	2,87	2,96	3,25
SAV0724	-1,79	-1,60	-3,35
SAV0725	NS	-1,54	-3,26
SAV0726	-2,11	-2,28	-1,76
SAV0727	-1,56	-1,56	-2,05
SAV0728	-1,61	-1,72	-1,84
SAV0729	-2,72	-2,69	-3,06
SAV0733	-3,61	-5,05	-9,92
SAV0734	NS	-1,72	-3,69
SAV0736	2,51	2,31	2,16
SAV0737	-1,52	-1,77	NS
SAV0740	3,82	3,72	3,46
SAV0745	-2,38	-2,24	-1,98
SAV0748	NS	NS	-1,53
SAV0749	-2,02	-2,28	-2,73
SAV0750	NS	NS	2,01
SAV0752	1,52	1,77	NS
SAV0756	1,73	2,44	2,21
SAV0757	2,10	2,35	2,73
SAV0766	1,70	NS	NS
SAV0767	2,18	1,77	2,00
SAV0769	NS	-1,55	NS
SAV0770	2,12	2,27	2,55
SAV0777	-1,97	-2,06	-3,08
SAV0780	1,63	NS	1,72
SAV0784	2,37	2,88	2,17
SAV0785	1,57	2,12	NS
SAV0787	NS	NS	-1,66
SAV0788	-2,19	-1,98	-2,74
SAV0789	-2,79	-2,46	-4,57
SAV0795	4,10	3,78	1,59
SAV0796	4,93	4,26	1,69
SAV0797	3,97	3,20	NS
SAV0798	5,25	4,02	1,79
SAV0799	NS	3,15	NS
SAV0800	NS	2,16	NS
SAV0801	2,44	2,67	2,05
SAV0802	NS	-1,57	-1,64
SAV0803	NS	-1,57	-1,62

SAV0804	4,12	3,47	2,60
SAV0807	4,21	3,32	2,08
SAV0808	2,25	2,08	NS
SAV0810	1,99	1,82	1,98
SAV0817	1,73	1,75	2,28
SAV0818	1,95	1,87	2,65
SAV0819	NS	NS	1,84
SAV0820	-2,92	-3,00	-4,55
SAV0821	-3,02	-3,05	-4,29
SAV0822	-3,14	-3,67	-3,18
SAV0823	-3,27	-3,71	-2,56
SAV0824	-2,27	-2,86	NS
SAV0825	-2,67	-2,13	-4,57
SAV0828	-3,13	-3,28	-2,85
SAV0830	-1,81	-2,11	-2,50
SAV0832	1,62	1,90	2,05
SAV0833	NS	-1,63	-1,84
SAV0834	NS	NS	1,85
SAV0837	NS	-1,79	-3,05
SAV0838	NS	NS	-1,64
SAV0839	NS	2,02	NS
SAV0840	1,53	NS	1,81
SAV0841	-1,65	-1,85	-1,56
SAV0842	2,20	2,32	2,04
SAV0843	1,99	1,71	1,65
SAV0850	NS	NS	-1,54
SAV0851	2,06	1,94	1,93
SAV0854	-2,51	-2,68	-3,32
SAV0857	-1,80	-1,71	-1,61
SAV0858	-2,19	-2,07	-2,10
SAV0859	-3,91	-3,95	-3,40
SAV0861	-2,18	NS	NS
SAV0862	-2,04	-2,18	NS
SAV0866	-1,97	-2,24	-2,01
SAV0867	-2,43	-2,62	-2,90
SAV0878	NS	-8,85	-3,77
SAV0899	NS	-3,66	NS
SAV0905	NS	-2,41	-2,62
SAV0906	NS	-2,54	-3,17
SAV0909	NS	-3,41	-3,89
SAV0910	NS	-2,14	-2,72
SAV0911	NS	-2,19	-2,39
SAV0912	NS	-2,96	-2,35
SAV0913	-3,50	-4,84	-3,13
SAV0914	NS	-2,43	-3,43

SAV0915	-1,54	NS	-2,04
SAV0916	3,86	2,78	2,38
SAV0917	6,38	4,47	3,51
SAV0918	-2,42	-2,02	-3,54
SAV0919	NS	NS	1,87
SAV0920	1,80	1,86	2,57
SAV0927	-2,16	-1,78	-1,78
SAV0936	-2,37	-1,92	-1,94
SAV0937	NS	NS	1,53
SAV0938	-1,87	-2,26	-3,19
SAV0939	-2,50	-1,92	-2,68
SAV0940	-2,30	-1,90	-2,55
SAV0941	NS	NS	1,54
SAV0943	-2,23	-1,71	-3,90
SAV0944	-2,16	-1,96	-3,30
SAV0953	NS	NS	-1,66
SAV0955	2,13	1,95	NS
SAV0963	-1,57	NS	NS
SAV0966	2,61	2,56	2,56
SAV0967	2,47	2,12	2,08
SAV0969	-2,57	-2,63	-2,07
SAV0971	1,56	1,69	NS
SAV0972	-2,21	-2,29	-2,24
SAV0973	-1,97	-2,04	-2,13
SAV0974	-1,56	-1,82	-1,82
SAV0977	3,53	3,28	2,95
SAV0978	2,73	3,00	2,82
SAV0979	NS	2,52	2,35
SAV0981	NS	2,16	2,88
SAV0982	-3,52	-3,19	-4,91
SAV0984	-1,85	-1,81	NS
SAV0985	-4,62	-3,83	-6,61
SAV0987	NS	-1,80	-2,48
SAV0990	2,51	1,77	NS
SAV0991	2,70	3,34	NS
SAV0998	2,18	3,61	2,04
SAV0999	3,38	4,13	3,46
SAV1000	2,36	2,62	3,59
SAV1004	1,66	2,22	1,64
SAV1006	1,56	1,81	NS
SAV1008	1,52	1,63	NS
SAV1012	-2,16	-2,19	-3,06
SAV1013	-3,32	-3,71	-9,96
SAV1014	-1,78	-1,76	-1,95
SAV1015	-1,75	-1,81	-2,64

SAV1017	NS	1,54	NS
SAV1019	1,63	1,70	2,03
SAV1020	2,07	2,27	2,72
SAV1021	2,13	1,96	2,63
SAV1022	NS	NS	-1,53
SAV1024	-1,69	-1,55	-2,00
SAV1025	-1,72	-1,72	-1,74
SAV1026	NS	NS	2,15
SAV1027	NS	NS	-5,54
SAV1029	NS	-1,53	1,85
SAV1030	NS	NS	2,04
SAV1031	-2,35	-2,30	-2,24
SAV1032	-4,36	-3,98	-7,96
SAV1033	3,37	4,18	NS
SAV1036	-2,79	-2,77	-3,59
SAV1037	-2,77	-2,51	NS
SAV1040	-2,76	-1,57	-1,95
SAV1041	-2,52	-2,27	-2,01
SAV1042	2,31	2,41	3,37
SAV1044	1,58	1,55	2,28
SAV1049	NS	NS	-1,60
SAV1050	NS	NS	1,97
SAV1052	3,17	2,82	2,51
SAV1055	3,92	4,57	5,46
SAV1056	4,01	4,86	5,38
SAV1058	-1,98	-2,61	-2,99
SAV1061	NS	-1,57	-1,82
SAV1062	NS	NS	1,54
SAV1064	-2,39	-2,47	-1,81
SAV1067	NS	NS	2,43
SAV1075	-1,63	-1,85	-2,92
SAV1076	NS	-1,76	-2,96
SAV1077	-2,52	-2,83	-5,43
SAV1078	-1,57	-1,78	-1,96
SAV1080	NS	NS	1,52
SAV1081	1,75	1,75	1,98
SAV1085	1,87	2,20	2,21
SAV1086	-1,87	-1,85	NS
SAV1087	-1,91	-1,85	NS
SAV1088	-2,30	-2,26	-2,24
SAV1089	1,69	1,52	2,21
SAV1092	7,17	9,01	10,36
SAV1103	2,96	3,61	2,62
SAV1104	-1,65	NS	-1,91
SAV1105	NS	NS	-1,56

SAV1107	-1,69	-1,76	-2,38
SAV1108	-2,13	-1,71	-2,36
SAV1109	NS	NS	1,85
SAV1112	-2,94	-3,00	-2,62
SAV1113	-1,63	NS	NS
SAV1115	-2,05	-2,24	-2,55
SAV1117	-1,72	-1,97	-1,95
SAV1118	1,66	1,89	1,70
SAV1127	1,53	1,80	2,01
SAV1131	NS	NS	-2,32
SAV1133	1,94	NS	NS
SAV1135	4,77	NS	NS
SAV1140	NS	NS	2,69
SAV1142	-2,10	-2,00	-1,87
SAV1143	2,44	2,24	2,76
SAV1152	NS	NS	1,52
SAV1154	NS	8,72	NS
SAV1155	3,93	5,18	4,36
SAV1156	2,11	3,37	3,16
SAV1157	NS	1,60	1,76
SAV1158	4,47	4,98	9,50
SAV1159	4,52	5,51	9,04
SAV1160	1,72	1,57	NS
SAV1161	1,76	1,77	NS
SAV1163	2,13	4,05	1,90
SAV1165	-1,55	NS	NS
SAV1166	NS	1,60	1,99
SAV1167	NS	NS	1,69
SAV1168	NS	2,46	3,71
SAV1170	NS	1,61	3,59
SAV1171	-2,03	-2,00	NS
SAV1172	-2,00	-1,88	-2,20
SAV1174	3,12	2,53	2,26
SAV1175	NS	NS	1,65
SAV1176	-1,72	-1,75	-1,82
SAV1177	1,96	2,02	2,52
SAV1178	-2,64	-1,95	-2,28
SAV1187	2,46	2,56	2,60
SAV1188	2,09	2,01	2,03
SAV1189	2,23	2,07	2,07
SAV1191	3,02	3,20	4,40
SAV1192	2,02	2,18	2,55
SAV1194	-2,07	NS	NS
SAV1206	1,92	1,88	3,93
SAV1207	-1,73	-1,64	-1,51

SAV1208	2,78	2,74	2,62
SAV1213	NS	1,56	NS
SAV1214	-2,69	-2,09	-2,59
SAV1215	-1,75	-1,68	-2,25
SAV1217	2,37	1,98	1,95
SAV1218	3,70	2,94	3,18
SAV1219	2,56	1,97	2,45
SAV1220	2,61	2,21	2,63
SAV1225	NS	1,58	1,62
SAV1226	2,05	1,80	2,17
SAV1228	1,74	1,70	2,56
SAV1235	3,15	2,90	4,51
SAV1236	2,65	2,41	4,31
SAV1242	NS	-1,53	NS
SAV1250	3,98	4,20	6,69
SAV1262	NS	NS	1,94
SAV1267	2,65	2,20	2,34
SAV1268	4,07	2,90	3,80
SAV1275	NS	-1,55	NS
SAV1277	1,88	1,86	2,16
SAV1278	3,94	3,64	4,58
SAV1279	2,86	2,64	3,40
SAV1280	2,00	1,77	2,42
SAV1282	1,83	1,79	NS
SAV1286	3,25	2,97	3,17
SAV1287	2,53	3,71	3,24
SAV1288	-1,92	-2,02	-1,94
SAV1289	NS	1,64	NS
SAV1290	2,01	3,32	2,54
SAV1291	3,00	3,95	3,72
SAV1292	2,16	2,08	2,16
SAV1293	3,83	3,58	3,94
SAV1299	4,71	4,77	7,82
SAV1305	1,57	1,57	NS
SAV1307	NS	NS	1,52
SAV1308	1,72	1,61	1,78
SAV1312	NS	NS	-2,91
SAV1314	NS	4,48	NS
SAV1316	1,82	2,31	1,76
SAV1318	3,25	3,87	2,42
SAV1319	3,99	4,61	2,67
SAV1320	1,78	1,81	NS
SAV1321	3,11	3,26	2,28
SAV1322	2,89	2,52	2,09
SAV1323	-2,04	-1,87	-2,73

SAV1325	2,40	2,81	2,46
SAV1326	-1,96	-1,85	-2,02
SAV1331	NS	-1,61	-2,44
SAV1332	-1,79	-1,92	-2,43
SAV1333	-2,41	-2,01	-3,38
SAV1337	NS	NS	2,84
SAV1338	2,48	2,79	5,21
SAV1341	5,66	6,66	4,42
SAV1344	-2,31	-2,47	NS
SAV1346	2,21	1,84	2,41
SAV1351	-1,56	NS	-1,58
SAV1352	NS	-1,52	-1,57
SAV1353	-2,95	-3,26	-5,05
SAV1358	1,73	1,97	2,03
SAV1359	-1,50	NS	NS
SAV1363	-3,10	-2,90	-4,56
SAV1364	NS	NS	-2,13
SAV1366	2,43	2,01	1,89
SAV1367	2,80	3,06	NS
SAV1377	-2,28	-1,88	-2,00
SAV1378	-2,04	-2,10	-2,36
SAV1379	NS	-1,65	NS
SAV1389	NS	NS	2,96
SAV1391	-2,13	-2,22	-3,30
SAV1398	NS	NS	-1,84
SAV1401	-6,20	-3,93	-7,96
SAV1403	-1,73	NS	-2,01
SAV1405	2,14	2,25	2,40
SAV1406	4,73	4,53	4,75
SAV1408	3,00	2,88	2,62
SAV1409	2,75	2,67	2,40
SAV1410	2,15	1,83	1,82
SAV1416	-6,72	-12,67	-17,77
SAV1417	-1,50	-1,70	NS
SAV1419	NS	NS	1,56
SAV1421	NS	1,60	3,10
SAV1422	NS	1,80	3,68
SAV1423	NS	2,30	3,49
SAV1424	NS	2,49	3,43
SAV1429	1,99	2,08	2,58
SAV1430	2,78	3,30	3,59
SAV1431	2,30	2,55	2,99
SAV1432	-2,22	-1,97	-1,57
SAV1437	NS	-1,69	NS
SAV1438	NS	2,26	NS

SAV1440	2,17	1,94	2,53
SAV1441	3,32	3,22	3,88
SAV1443	NS	NS	2,32
SAV1446	4,35	3,97	6,42
SAV1447	3,47	3,53	5,16
SAV1448	3,40	3,28	5,58
SAV1451	3,20	2,92	4,10
SAV1453	3,05	3,05	4,07
SAV1456	NS	1,55	1,61
SAV1457	3,79	3,14	4,20
SAV1458	2,53	2,46	2,59
SAV1459	1,61	1,76	1,67
SAV1460	-1,55	-1,92	NS
SAV1462	1,78	1,72	2,00
SAV1463	2,42	2,31	2,60
SAV1472	NS	1,54	NS
SAV1480	NS	1,71	2,72
SAV1482	NS	NS	1,88
SAV1483	2,00	1,89	2,69
SAV1485	-2,23	-2,20	-1,63
SAV1486	1,58	NS	NS
SAV1487	2,01	1,88	1,67
SAV1488	2,96	2,65	2,44
SAV1489	2,49	2,03	2,04
SAV1494	3,20	2,67	2,89
SAV1498	-1,92	-1,73	NS
SAV1499	1,82	1,74	2,12
SAV1504	-2,24	-2,10	-2,14
SAV1505	1,70	2,17	1,66
SAV1506	-1,60	-1,52	-1,54
SAV1510	-2,08	-2,25	-2,00
SAV1513	NS	NS	1,60
SAV1522	6,06	5,54	6,98
SAV1525	3,02	3,48	3,45
SAV1529	NS	NS	1,63
SAV1531	2,35	2,90	2,30
SAV1532	2,07	2,42	2,26
SAV1533	-1,93	-1,84	-1,67
SAV1534	-1,61	-1,59	NS
SAV1535	3,24	3,28	9,57
SAV1536	4,49	4,52	14,12
SAV1538	-1,95	-1,63	-2,42
SAV1541	NS	6,32	NS
SAV1546	1,89	1,70	1,61
SAV1548	2,51	2,51	2,74

SAV1550	1,72	1,66	NS
SAV1554	NS	NS	1,60
SAV1556	NS	NS	1,56
SAV1557	2,60	2,12	2,39
SAV1558	2,50	2,15	2,31
SAV1559	1,69	NS	NS
SAV1560	1,75	NS	NS
SAV1563	1,91	1,97	1,97
SAV1572	5,39	4,51	3,97
SAV1573	1,54	NS	NS
SAV1584	-1,65	NS	-1,95
SAV1590	2,80	2,49	3,88
SAV1591	NS	NS	1,54
SAV1592	NS	NS	1,68
SAV1593	1,64	NS	2,05
SAV1594	2,34	1,75	2,71
SAV1595	3,92	3,02	4,65
SAV1597	1,76	NS	2,12
SAV1600	-2,11	-1,91	-1,80
SAV1603	NS	2,13	1,52
SAV1604	-1,73	-2,12	-2,79
SAV1605	NS	NS	-1,54
SAV1607	2,76	2,48	2,09
SAV1609	NS	NS	-1,55
SAV1612	2,17	1,73	2,50
SAV1613	1,75	NS	2,16
SAV1615	2,29	2,54	1,78
SAV1616	3,15	3,53	2,71
SAV1617	2,34	2,70	2,06
SAV1619	1,83	1,63	1,61
SAV1620	1,64	1,68	NS
SAV1623	NS	1,87	NS
SAV1624	2,67	2,25	3,32
SAV1626	-2,04	-1,76	-2,48
SAV1633	3,14	3,20	2,73
SAV1636	2,23	2,26	1,70
SAV1638	-1,88	-1,57	-2,20
SAV1643	NS	-1,72	-1,75
SAV1649	NS	1,78	1,63
SAV1650	2,32	2,73	2,92
SAV1652	-1,81	-1,61	-1,66
SAV1653	-2,46	NS	-2,89
SAV1654	1,60	1,67	1,78
SAV1661	-4,00	-3,61	-5,57
SAV1665	-1,73	-1,67	-2,17

SAV1666	-2,30	-2,32	-2,25
SAV1676	1,91	1,92	2,23
SAV1681	-1,88	-1,77	-2,16
SAV1689	2,88	2,54	3,23
SAV1701	2,03	1,84	1,92
SAV1707	-3,03	-3,23	-3,05
SAV1710	-1,62	-1,63	NS
SAV1712	1,54	1,62	1,95
SAV1715	2,39	2,04	2,19
SAV1716	2,56	2,11	2,33
SAV1717	3,51	3,15	2,43
SAV1723	NS	NS	-1,85
SAV1725	-1,92	-1,77	-2,45
SAV1727	-2,02	-2,01	-2,41
SAV1728	NS	NS	2,11
SAV1731	NS	NS	-1,87
SAV1738	2,88	2,43	3,55
SAV1739	-1,55	-1,82	NS
SAV1741	2,02	1,70	3,15
SAV1742	3,72	2,99	5,49
SAV1743	1,74	1,57	2,83
SAV1746	-1,80	NS	-1,67
SAV1747	-2,21	-1,90	-2,15
SAV1749	NS	-1,56	-2,15
SAV1750	NS	NS	1,61
SAV1751	-2,92	-2,24	-2,77
SAV1752	3,35	2,76	3,06
SAV1753	5,48	4,92	5,14
SAV1754	-1,69	-1,63	-1,91
SAV1755	-2,09	-2,26	-2,34
SAV1756	-1,60	-2,00	-1,84
SAV1759	NS	NS	1,76
SAV1761	-2,04	-2,11	-1,99
SAV1762	NS	NS	1,63
SAV1763	NS	1,50	1,94
SAV1765	NS	NS	-1,55
SAV1773	-2,77	-2,69	-2,67
SAV1774	-2,45	-2,27	-2,14
SAV1775	3,78	4,68	4,29
SAV1776	3,64	4,41	4,69
SAV1777	1,72	1,74	2,00
SAV1778	1,62	1,89	2,12
SAV1779	NS	1,69	1,87
SAV1780	2,67	3,42	4,52
SAV1781	1,71	1,86	1,89

SAV1782	-1,74	NS	NS
SAV1783	-2,15	-1,67	-1,63
SAV1784	-2,01	-1,98	-1,67
SAV1785	NS	NS	2,86
SAV1789	2,27	2,50	1,99
SAV1793	NS	-1,60	NS
SAV1795	-1,87	NS	-2,47
SAV1798	NS	2,40	2,62
SAV1799	3,12	2,32	1,63
SAV1800	2,96	2,23	1,64
SAV1804	3,10	2,65	1,88
SAV1805	3,44	2,93	2,19
SAV1807	3,48	3,04	2,69
SAV1808	-1,99	-1,94	-2,34
SAV1815	-1,57	NS	NS
SAV1818	NS	1,93	NS
SAV1821	3,02	3,17	2,62
SAV1822	1,68	1,70	NS
SAV1823	2,21	2,32	2,02
SAV1836	-1,60	-1,79	NS
SAV1837	-2,67	-2,49	-1,66
SAV1843	4,71	3,93	3,65
SAV1844	NS	1,55	NS
SAV1845	5,99	6,33	5,83
SAV1846	4,38	4,39	3,17
SAV1847	1,51	1,65	2,65
SAV1848	3,08	3,32	3,71
SAV1852	-2,45	-2,34	-2,86
SAV1853	2,78	2,61	2,90
SAV1854	2,35	2,34	2,42
SAV1856	-1,60	-1,56	-1,74
SAV1858	NS	2,39	NS
SAV1859	-2,44	-2,48	-2,60
SAV1861	NS	1,77	NS
SAV1862	4,46	5,41	5,66
SAV1863	3,86	4,99	4,87
SAV1865	NS	1,63	1,72
SAV1867	-1,79	NS	-1,76
SAV1868	-1,54	NS	-1,68
SAV1869	-1,73	-1,62	-2,31
SAV1870	NS	NS	1,53
SAV1872	1,69	2,00	1,80
SAV1874	2,61	4,61	6,05
SAV1875	-2,52	-2,16	-2,55
SAV1876	-1,91	-2,00	-1,90

SAV1881	-2,47	-2,64	-3,59
SAV1882	-2,38	-2,59	-3,60
SAV1886	1,66	2,66	3,76
SAV1887	4,19	8,03	11,21
SAV1889	-1,78	-1,80	-1,96
SAV1891	3,06	2,66	3,17
SAV1893	3,32	5,52	5,23
SAV1894	NS	NS	1,59
SAV1895	-1,64	-1,61	-1,50
SAV1896	NS	1,90	3,21
SAV1897	NS	2,04	3,95
SAV1898	NS	-1,56	NS
SAV1903	4,40	4,84	4,69
SAV1907	1,64	1,72	2,53
SAV1910	NS	1,79	1,75
SAV1916	-3,30	-3,51	-4,88
SAV1917	-1,53	NS	NS
SAV1918	-1,88	-1,57	-2,26
SAV1919	NS	NS	-1,62
SAV1922	-2,19	-2,23	-2,63
SAV1923	-1,61	NS	-1,73
SAV1924	-1,79	NS	-1,70
SAV1926	1,90	1,72	1,99
SAV1927	2,05	2,00	2,09
SAV1928	NS	NS	1,53
SAV1929	3,14	4,11	2,72
SAV1930	NS	1,59	1,63
SAV1931	1,99	1,91	2,56
SAV1932	2,09	2,04	2,86
SAV1933	NS	NS	1,79
SAV1935	-2,44	-1,84	-1,60
SAV1938	3,19	4,75	3,47
SAV1939	3,73	5,46	2,68
SAV1940	1,67	1,95	1,91
SAV1941	NS	1,52	NS
SAV1942	6,66	7,00	4,63
SAV1943	-1,79	-1,75	-2,39
SAV1947	-2,39	-1,87	-2,88
SAV1951	NS	-2,62	NS
SAV1970	NS	-4,09	-3,42
SAV1971	NS	-6,13	-4,27
SAV1973	NS	-4,97	-16,10
SAV1975	NS	-29,75	-17,49
SAV1990	-3,28	-3,21	-3,27
SAV1991	-3,11	-3,11	-2,81

SAV1992	NS	NS	-1,50
SAV1993	-1,90	-2,16	-1,69
SAV1995	NS	-1,64	-1,99
SAV1996	NS	NS	-1,82
SAV1997	NS	NS	-2,16
SAV1998	2,40	2,12	1,89
SAV1999	4,06	3,40	2,92
SAV2000	4,84	3,67	3,77
SAV2001	3,16	2,31	2,09
SAV2010	NS	NS	-1,75
SAV2012	NS	1,57	-1,54
SAV2015	5,42	4,11	1,82
SAV2016	4,09	3,30	NS
SAV2017	4,91	4,22	1,72
SAV2018	4,35	3,97	1,67
SAV2021	-2,47	-2,64	-2,49
SAV2022	-2,15	-2,18	-1,81
SAV2023	-3,33	-3,50	-5,85
SAV2024	-3,23	-3,59	-5,40
SAV2025	NS	-2,08	-2,89
SAV2026	NS	-1,87	-2,13
SAV2027	NS	1,52	NS
SAV2031	-2,63	-2,23	-2,42
SAV2032	NS	1,54	NS
SAV2034	-1,82	NS	-1,51
SAV2040	NS	-1,55	-1,65
SAV2046	NS	NS	-1,67
SAV2049	NS	NS	1,79
SAV2050	2,07	2,69	2,94
SAV2051	NS	2,00	1,95
SAV2052	NS	1,88	1,51
SAV2055	NS	NS	-2,56
SAV2063	1,70	1,52	1,79
SAV2068	NS	NS	-1,76
SAV2069	NS	NS	-2,30
SAV2074	-2,08	-2,12	-2,64
SAV2084	-2,56	-2,60	-2,48
SAV2085	-1,85	-1,85	-2,59
SAV2086	1,57	1,53	1,63
SAV2088	NS	NS	-1,53
SAV2089	-1,65	-1,89	-1,91
SAV2090	NS	NS	2,04
SAV2094	-3,17	-3,13	-2,08
SAV2095	NS	NS	4,85
SAV2101	-1,81	NS	NS

SAV2110	NS	-1,62	NS
SAV2114	NS	-1,69	-1,80
SAV2115	NS	-1,66	NS
SAV2116	-1,67	-2,06	NS
SAV2123	NS	-1,52	NS
SAV2126	-1,58	-1,63	-1,58
SAV2130	-2,66	-2,88	-1,90
SAV2135	-1,63	-1,85	-1,87
SAV2140	2,53	3,29	3,00
SAV2142	-2,10	-2,00	-1,81
SAV2143	-2,79	-2,61	-2,88
SAV2144	-3,72	-4,24	-3,04
SAV2147	3,79	4,00	5,09
SAV2150	1,69	1,58	NS
SAV2151	2,35	2,14	NS
SAV2157	NS	-2,49	-2,27
SAV2162	2,59	2,41	2,46
SAV2163	-1,56	-1,55	-1,78
SAV2165	NS	-1,57	NS
SAV2166	-1,68	-1,77	NS
SAV2167	-2,16	-1,92	NS
SAV2168	NS	-1,67	-1,95
SAV2169	-1,68	-2,09	-2,24
SAV2170	-1,54	-1,95	-2,06
SAV2172	-1,66	-1,59	-2,34
SAV2173	3,03	3,30	3,60
SAV2180	-1,66	-1,96	-1,89
SAV2181	1,51	NS	1,92
SAV2183	2,32	1,74	NS
SAV2185	-2,00	-2,37	-2,75
SAV2197	-1,97	-1,88	-2,44
SAV2198	2,65	2,29	NS
SAV2199	2,53	2,36	1,57
SAV2200	3,74	4,22	3,99
SAV2201	3,70	4,57	3,07
SAV2204	NS	NS	1,91
SAV2206	2,49	2,10	1,81
SAV2208	NS	-2,51	-3,11
SAV2209	3,64	3,47	2,95
SAV2210	2,21	2,08	NS
SAV2211	4,67	4,15	3,62
SAV2212	3,03	3,29	3,58
SAV2213	2,69	3,16	2,70
SAV2214	2,72	2,19	2,37
SAV2215	1,74	1,79	1,89

SAV2216	2,24	2,44	2,04
SAV2220	1,57	NS	1,65
SAV2252	-1,90	-1,73	-1,55
SAV2253	NS	-1,53	NS
SAV2255	-2,10	-1,81	-2,39
SAV2256	-1,85	-1,68	NS
SAV2257	-1,65	-1,56	-1,93
SAV2258	-2,07	-1,62	-1,68
SAV2259	-1,85	-2,14	-1,82
SAV2260	NS	-1,59	-1,56
SAV2261	1,88	1,58	2,24
SAV2263	-1,98	-1,58	-1,78
SAV2264	-2,09	-1,62	-1,68
SAV2267	2,00	2,07	NS
SAV2283	-1,68	-1,72	-1,65
SAV2284	2,40	1,93	NS
SAV2287	-2,49	-2,49	-2,63
SAV2296	-1,65	NS	-1,92
SAV2297	2,33	2,54	1,81
SAV2298	1,94	2,04	1,71
SAV2300	-5,29	-5,62	-4,95
SAV2301	-2,36	-2,52	-2,66
SAV2302	-3,01	-2,85	-2,68
SAV2303	-2,32	-2,16	-3,31
SAV2304	1,85	2,57	3,55
SAV2305	-1,76	-1,80	-1,99
SAV2309	NS	NS	-1,57
SAV2310	NS	NS	2,24
SAV2311	1,59	NS	2,11
SAV2312	1,79	1,90	1,57
SAV2314	2,46	2,57	3,35
SAV2315	3,12	3,00	2,34
SAV2316	-2,46	-2,77	-2,78
SAV2317	-2,31	-2,07	-2,31
SAV2318	-2,20	-1,94	-1,91
SAV2319	-2,05	-1,99	-1,95
SAV2320	-2,39	-2,04	-2,22
SAV2321	-2,32	-1,74	-2,46
SAV2322	-2,33	NS	1,81
SAV2326	-2,64	-2,67	-2,34
SAV2327	-2,70	-2,55	-2,85
SAV2328	-2,97	-3,40	-4,21
SAV2329	-2,28	-2,18	-3,29
SAV2332	NS	1,86	NS
SAV2334	-2,83	-3,02	-2,51

SAV2335	1,60	1,65	1,91
SAV2336	-3,06	-3,15	-2,83
SAV2338	-1,89	-1,92	-2,10
SAV2339	-3,39	-3,83	-3,86
SAV2342	-2,41	-2,89	-3,52
SAV2343	-3,58	-3,88	-4,58
SAV2344	-1,81	-1,91	-2,04
SAV2345	-1,95	-1,96	NS
SAV2347	NS	NS	-1,91
SAV2348	1,63	1,85	1,99
SAV2349	NS	1,64	NS
SAV2350	NS	NS	-1,63
SAV2352	1,83	1,81	1,60
SAV2353	2,14	2,32	2,09
SAV2362	1,59	NS	1,54
SAV2365	-1,61	-1,87	-1,62
SAV2366	-1,77	-2,05	-2,02
SAV2368	2,40	2,77	1,88
SAV2370	-1,66	-1,66	NS
SAV2371	-1,82	-2,60	-2,06
SAV2372	-2,17	-2,91	-2,48
SAV2373	-3,28	-3,34	-2,75
SAV2374	NS	1,77	NS
SAV2375	2,64	2,42	2,77
SAV2376	3,19	3,06	3,48
SAV2381	2,36	2,27	2,00
SAV2383	-2,81	-2,23	-2,17
SAV2385	2,18	2,71	2,35
SAV2386	2,31	1,91	NS
SAV2387	1,53	1,69	1,58
SAV2393	-1,98	-1,88	-2,47
SAV2395	-1,93	NS	NS
SAV2402	-1,63	NS	NS
SAV2403	-2,81	-2,94	-3,11
SAV2404	NS	2,01	NS
SAV2406	1,71	1,68	1,56
SAV2407	2,11	2,32	1,81
SAV2408	2,03	1,94	1,69
SAV2409	3,63	3,86	2,49
SAV2410	1,79	2,02	NS
SAV2414	NS	1,78	2,86
SAV2415	NS	NS	1,52
SAV2417	-1,97	-1,98	-1,84
SAV2422	2,70	2,96	1,84
SAV2423	NS	2,10	2,03

SAV2430	-2,35	-2,06	-2,75
SAV2431	-3,05	-3,32	-2,56
SAV2432	NS	1,74	2,97
SAV2433	2,47	3,82	6,89
SAV2434	-2,61	-2,62	-3,88
SAV2436	-2,21	-1,70	-2,52
SAV2438	-1,71	-2,05	-1,90
SAV2439	NS	1,56	NS
SAV2440	-2,07	-2,03	-3,48
SAV2441	NS	1,55	NS
SAV2443	-1,73	-1,59	-1,67
SAV2444	-1,77	NS	-1,77
SAV2449	2,90	2,94	3,54
SAV2450	-1,94	-1,69	-3,04
SAV2451	-1,98	-2,02	-1,78
SAV2453	-2,44	-2,68	-2,18
SAV2454	NS	-1,55	NS
SAV2455	-1,73	NS	-1,81
SAV2457	2,54	2,57	2,19
SAV2458	4,91	6,23	4,10
SAV2459	-2,08	-2,12	-1,65
SAV2460	-4,44	-3,67	-4,29
SAV2462	-2,01	-1,97	-1,97
SAV2463	-1,97	-2,06	-2,06
SAV2464	-2,13	-2,14	-2,17
SAV2465	-2,22	-2,84	-2,75
SAV2466	NS	-1,63	-1,67
SAV2467	1,81	1,56	NS
SAV2468	NS	-1,92	-1,99
SAV2469	-1,55	-2,10	-2,45
SAV2470	-2,89	-3,80	-4,63
SAV2471	-3,55	-4,02	-4,33
SAV2472	-3,93	-4,27	-4,20
SAV2473	-2,30	-2,04	-4,33
SAV2474	-2,06	-1,93	-3,03
SAV2477	3,27	3,93	4,82
SAV2479	-2,12	-2,12	-1,52
SAV2482	3,17	2,83	3,32
SAV2483	4,13	3,65	4,09
SAV2484	3,10	3,14	2,91
SAV2485	2,05	2,47	1,66
SAV2486	2,60	2,21	2,56
SAV2487	2,98	2,85	2,63
SAV2488	4,75	4,23	4,09
SAV2489	2,56	2,44	2,76

SAV2490	1,74	1,85	1,91
SAV2491	-1,57	NS	NS
SAV2492	NS	1,52	NS
SAV2496	NS	-3,77	-3,50
SAV2497	NS	-4,97	-3,90
SAV2508	NS	1,99	3,26
SAV2509	NS	2,33	4,40
SAV2510	NS	NS	2,03
SAV2511	-1,50	-1,55	NS
SAV2512	-2,23	-2,59	-3,58
SAV2513	-1,92	-1,62	NS
SAV2514	3,92	4,10	3,79
SAV2517	-2,12	-2,05	-1,60
SAV2518	-1,74	-3,07	-2,42
SAV2519	-1,81	-3,04	-2,61
SAV2520	-2,37	-2,68	-3,62
SAV2521	-2,89	-2,30	-2,21
SAV2522	-1,94	-1,68	-1,62
SAV2526	2,30	2,93	NS
SAV2527	-1,59	-1,58	-2,40
SAV2529	-1,50	NS	-1,73
SAV2530	-1,96	-2,11	-1,94
SAV2532	-1,95	-2,10	NS
SAV2533	-3,14	-3,34	-2,20
SAV2534	-2,46	-2,49	-2,19
SAV2536	-1,53	NS	NS
SAV2537	-2,11	NS	-2,06
SAV2539	1,51	1,88	1,67
SAV2542	-2,66	-2,29	-2,32
SAV2543	-2,49	-1,93	-3,59
SAV2544	-1,56	NS	NS
SAV2549	-1,58	-2,38	-2,39
SAV2551	-2,77	-4,36	-7,75
SAV2552	NS	NS	2,06
SAV2553	1,91	1,97	2,07
SAV2555	2,61	3,98	5,68
SAV2559	-2,34	-2,03	-2,32
SAV2560	-2,11	-1,84	-2,43
SAV2563	NS	-1,66	NS
SAV2566	NS	NS	1,95
SAV2568	4,52	4,19	6,71
SAV2570	-3,97	-3,33	-4,58
SAV2573	NS	1,89	NS
SAV2576	NS	NS	2,00
SAV2580	-1,77	-1,70	NS

SAV2581	-2,33	-2,32	-2,31
SAV2586	1,78	1,72	1,70
SAV2588	-3,25	-3,59	-3,54
SAV2589	NS	NS	2,21
SAV2590	2,36	2,60	3,41
SAV2592	-2,10	-2,05	-1,79
SAV2600	-2,36	-2,31	-1,68
SAV2601	-2,36	-2,73	-2,68
SAV2602	-2,67	-2,86	-2,80
SAV2603	-2,02	-1,69	-5,52
SAV2604	-2,78	-2,00	-5,65
SAV2605	-2,45	-2,47	-2,58
SAV2606	2,85	2,43	3,13
SAV2608	-3,12	-2,98	-2,72
SAV2611	NS	1,76	NS
SAV2614	-1,55	-1,79	-1,51
SAV2620	NS	NS	1,74
SAV2622	1,50	NS	1,59
SAV2623	-1,80	-1,79	-1,55
SAV2624	3,70	2,63	4,66
SAV2625	3,24	2,44	4,00
SAV2626	3,95	2,83	5,33
SAV2629	-1,63	NS	-2,04
SAV2639	1,73	1,62	1,60
SAV2640	2,11	2,31	1,80
SAV2641	-1,78	-1,85	-2,01
SAV2643	1,85	1,72	1,79
SAV2646	2,35	2,09	1,87
SAV2647	-1,53	-1,77	-2,10
SAV2648	-1,58	-2,01	-2,08
SAV2649	-1,96	-2,38	-2,18
SAV2654	1,51	NS	1,87
SAV2655	-4,64	-4,34	-7,49
SAV2656	-1,60	-1,67	NS
SAV2657	-1,82	-1,81	-1,57
SAV2658	-3,95	-4,46	-5,16
SAV2659	-2,30	-2,16	-1,80
SAV2660	NS	-1,93	NS
SAV2661	-2,16	-2,07	-1,87
SAV2670	NS	NS	-1,82
SAV2674	NS	NS	-2,10
SAV2677	NS	-1,51	-2,28
SAV2681	NS	1,76	NS
SAV2682	2,92	2,99	2,32
SAV2685	-1,51	NS	NS

SAV2686	-2,12	-1,66	-1,65
SAV2687	-2,56	-2,15	-1,91
SAV2689	-1,77	NS	1,64
SAV2691	NS	NS	-1,81
SAV2693	-4,72	-4,99	-3,96
SAV2694	-3,50	-3,67	-4,23
SAV2695	-2,67	-3,04	-3,47
SAV2696	-1,94	-1,50	-1,72
SAV2697	-2,29	-2,53	NS
SAV2698	-1,85	-1,52	NS
SAV2699	1,51	1,87	1,70
SAV2700	-4,48	-5,46	-6,79
SAV2703	NS	NS	2,09
SAV2705	-1,88	-1,90	-1,73
SAV2706	-2,93	-2,95	-2,58
SAV2707	-3,15	-3,08	-3,17
SAV2708	-1,53	NS	NS
SAV2709	1,51	1,72	NS
SAVrRNA02	-1,85	NS	NS
SAVtRNA02	-2,06	-2,11	-2,74
SAVtRNA03	-2,95	-2,76	-4,19
SAVtRNA04	-4,96	-5,14	-10,17
SAVtRNA05	-3,98	-3,59	-7,60
SAVtRNA06	-4,31	-3,54	-7,48
SAVtRNA07	-4,68	-4,32	-8,10
SAVrRNA06	NS	NS	-2,15
SAVrRNA07	NS	NS	-2,34
SAVtRNA08	-3,75	-5,23	-4,78
SAVtRNA09	-4,03	-4,78	-8,17
SAVrRNA09	NS	NS	-2,28
SAVtRNA10	-5,29	-5,75	-10,36
SAVtRNA11	-6,45	-8,13	-11,26
SAVrRNA13	-1,94	NS	-2,36
SAVtRNA15	NS	NS	-1,60
SAVtRNA17	-2,15	NS	-2,03
SAVtRNA18	-4,71	-3,18	-9,04
SAVtRNA19	-3,62	-2,29	-7,30
SAVtRNA20	-4,83	-3,68	-8,16
SAVtRNA21	-5,66	-3,80	-10,70
SAVtRNA22	-5,88	-3,45	-9,16
SAVtRNA23	-3,72	-2,89	-5,26
SAVtRNA25	-1,78	-2,15	-2,63
SAVtRNA26	-2,45	-2,41	-4,24
SAVtRNA27	-2,05	-2,86	-4,59
SAVtRNA28	-2,21	-3,31	-4,53

SAVtRNA29	-2,40	-3,45	-4,75
SAVtRNA30	-2,25	-3,15	-4,71
SAVtRNA31	-2,24	-3,35	-5,44
SAVtRNA32	-2,99	-4,46	-6,56
SAVtRNA33	-2,45	-3,42	-7,88
SAVtRNA34	-3,08	-3,70	-5,64
SAVtRNA35	-2,53	-4,14	-2,82
SAVtRNA36	-2,58	-2,78	-4,32
SAVtRNA37	-2,49	-3,12	-4,17
SAVtRNA38	-2,58	-3,18	-4,08
SAVtRNA39	-2,29	-3,28	-5,01
SAVtRNA40	-2,19	-2,52	-4,43
SAVtRNA41	-2,33	-2,80	-3,57
SAVtRNA42	-2,70	-3,66	-4,07
SAVtRNA43	-2,71	-3,86	-5,32
SAVtRNA44	-2,62	-3,64	-3,60
SAVtRNA45	-2,53	-3,35	-2,61
SAVtRNA46	-2,53	-2,89	-3,88
SAVtRNA47	-2,09	-2,55	-3,61
SAVtRNA48	-1,61	-1,79	-2,96
SAVtRNA49	-2,51	-2,71	-2,07
SAVtRNA50	-3,81	-5,01	NS
SAVtRNA51	-2,59	-3,70	-3,07
SAVtRNA52	-3,84	-5,31	-6,63
SAVtRNA53	-3,00	-2,48	-4,58
SAVtRNA54	-2,39	-2,12	-4,45
SAVtRNA55	-2,21	-2,53	-2,72
SAVtRNA56	-2,66	-3,19	-3,49
SAVtRNA57	-3,34	-3,78	-3,46
SAVtRNA58	-3,76	-5,68	-3,37
SAVtRNA59	-3,40	-4,45	-3,75
sbi	3,13	3,36	5,04
scrA	-2,11	-2,91	-3,30
scrR	2,05	2,03	2,13
sdhA	NS	NS	-1,62
sdhB	NS	NS	-1,75
sdhC	-2,26	-2,38	-3,69
sdrD	NS	NS	1,51
sdrE	2,46	2,54	2,44
sec3	2,12	2,38	1,67
secA	1,80	1,91	2,45
secE	2,44	2,18	2,53
secF	1,83	1,90	2,10
secG	-2,90	-3,30	-3,28
seg	NS	2,35	NS

sel	1,84	1,89	NS
sem	-1,84	NS	NS
sen	NS	3,90	NS
serA	NS	NS	-2,26
serS	NS	NS	-2,67
set10	2,37	2,60	2,25
set11	1,97	2,70	2,24
set12	NS	3,05	NS
sigB	2,08	1,73	1,56
sirA	NS	NS	-2,48
sirB	NS	-1,66	-2,54
sirC	-3,76	-4,34	-4,68
smc	3,80	3,65	4,68
smpB	NS	NS	1,51
spa	2,66	3,22	3,65
spoIIIE	2,36	1,99	2,53
spoVG	NS	-1,94	-2,37
spsA	NS	NS	1,61
spsB	1,52	1,84	2,86
spxA	1,69	1,68	1,72
srrA	-1,52	-1,75	-1,54
srrB	1,63	NS	1,85
srtA	2,64	3,05	2,53
ssaA	-2,65	-2,75	NS
sspB	2,03	1,99	2,09
sspC	2,43	2,52	2,49
sucA	1,57	1,50	1,59
tag	NS	NS	-1,74
tagA	-1,52	NS	-1,71
tagB	2,04	1,96	2,33
tagD	2,12	1,96	2,74
tagG	-1,65	-1,70	-1,51
tagX	2,67	2,41	3,08
tcaA	3,32	4,68	10,19
tcaB	-2,34	-2,61	-1,79
tcaR	2,95	3,21	4,07
tdk	-1,74	-2,05	-1,69
tgt	NS	-1,81	NS
thiD	NS	-1,57	NS
thiE	-1,87	-2,01	NS
thrB	NS	NS	-2,09
thrC	NS	NS	-1,71
thrS	NS	NS	-1,91
thyA	1,73	NS	2,05
tig	4,12	4,15	4,50

tnp (NC_002758 1934745..1935296)	3,22	3,06	2,46
tnp (NC_002758 36410..37135)	1,85	1,81	NS
tnp (NC_002758 41756..42481)	1,85	1,79	NS
tnp (NC_002758 460207..461448)	-1,96	-2,22	-3,10
tnp (NC_002758 70144..70572)	-2,85	-2,44	-2,29
tnp (NC_002758 70633..71037)	-2,16	-2,34	-1,57
tnpB (NC_002758 1764405..1766348)	1,75	1,63	NS
tnpB (NC_002758 58111..60054)	1,78	1,64	NS
tnpC (NC_002758 1764021..1764449)	4,42	3,69	3,14
tnpC (NC_002758 57727..58155)	4,25	3,65	2,95
topB	3,10	2,89	2,95
traP	-1,54	NS	-1,84
treP	-2,54	-3,32	-4,29
trmB	NS	NS	-1,73
trmE	NS	NS	1,72
trpC	NS	3,57	NS
trpD	NS	2,29	NS
trpF	NS	3,81	NS
trpG	NS	3,25	NS
trpS	NS	NS	-1,61
truA	1,71	NS	1,82
truB	NS	NS	1,91
truncated-arlR	1,52	NS	NS
truncated-hlb	1,91	1,83	2,85
truncated-kdpA	-2,62	-2,59	-6,84
truncated-mapW	2,89	4,35	2,38
truncated-radC (NC_002758 1767498..1768103)	NS	-2,12	-2,53
truncated-radC (NC_002758 61204..61572)	-2,68	NS	-2,75
truncated-tnp	-1,63	NS	NS
tsf	1,98	2,33	2,50
tst	NS	2,13	NS
tyrS	NS	NS	1,85
ubiE	2,06	2,01	1,92
udk	2,34	2,11	2,74
uhpT (NC_002758 253202..254632)	-3,03	-3,27	-3,27
ulaA	NS	-1,93	-2,23
ung	-1,70	-1,69	NS
uppP	-1,67	NS	1,82
uppS	2,40	3,15	5,70
ureA	NS	-2,00	-1,68
ureB	NS	NS	-1,55
uvrC	3,17	2,93	4,06
veg	-1,72	-2,18	-1,62

vga	3,24	3,23	3,02
vicK	2,17	1,80	2,28
vicR	2,14	1,89	2,39
vraA	-2,70	-3,75	-4,76
vraB	-2,05	-2,33	-1,71
vraC	-2,69	-2,51	-1,60
vraD	NS	NS	2,31
vraE	NS	1,82	3,81
vraG	2,34	2,27	2,54
vraR	1,69	1,98	2,65
vraS	2,44	3,03	4,37
xerD	NS	NS	1,66
xprT	NS	1,55	-1,50
xseA	3,27	3,09	3,47
yent1	NS	4.51	NS
yent2	NS	6,43	NS
yjbM	1,90	2,50	2,01
yrhB	NS	NS	5,09

Table S3: Changes in the *S. aureus* Mu50 gene expression by RNA sequence analysis after 2h treatment with VAN compared to the untreated control (CTRL) for genes previously reported to be differentially regulated in glycopeptide resistant strains or after glycopeptide treatment. The RNA sequence data are presented as the mean fold-change of 3 separate experiments. Data that met criteria for differentially-expressed genes (P value <0.05; >1.5-fold change) are included. Red values indicate that genes are downregulated and green values indicate that genes are upregulated in these treatment conditions compared to the untreated control.

Feature ID	VAN vs CTRL Fold Change (normalized values)	Reference
aldA	1.60	(1)
clpX	2.55	(1)
csbD	1.83	(2)
dnaK	5.37	(1)
fbp	1.99	(1)
fmt	2.20	(1-2)
folD	1.51	(3)
glpQ	1.55	(2)
lytM	2.01	(3)
malR	-2.13	(1)
msrR	1.80	(4)
murF	1.55	(1)
nadE	2.59	(1)
Pbp2	3.54	(1)
prsA	7.85	(1-2)

purC	-1.70	(1)
purF	-1.70	(1)
pyrAA	-1.71	(1)
pyrR	-4.16	(1)
qoxB	-1.85	(1)
qoxC	-2.54	(1)
recU	3.49	(1)
ribA	2.17	(1)
ribD	1.81	(1)
SarA	4.03	(2)
SAV0580	-1.50	(1)
SAV1000	2.62	(1)
SAV1874	4.61	(4)
SAV1886	2.66	(2)
SAV2530	-2.11	(1)
sdhC	-2.38	(1)
secA	1.91	(1)
spsB	1.84	(1)
tcaA	4.68	(1-2)
tdk	-2.05	(1)
tig	4.15	(1)
vraR	1.98	(1-3
vraS	3.03	(1-3)

References:

1 Muthaiyan, A., Silverman, J. A., Jayaswal, R. K. & Wilkinson, B. J. Transcriptional profiling reveals that daptomycin induces the *Staphylococcus aureus* cell wall stress

stimulon and genes responsive to membrane depolarization. *Antimicrobial agents and chemotherapy* **52**, 980-990, doi:10.1128/aac.01121-07 (2008).

2 McAleese, F. *et al.* Overexpression of genes of the cell wall stimulon in clinical isolates of *Staphylococcus aureus* exhibiting vancomycin-intermediate- *S. aureus*-type resistance to vancomycin. *Journal of bacteriology* **188**, 1120-1133, doi:10.1128/jb.188.3.1120-1133.2006 (2006).

3 Rose, W. E., Fallon, M., Moran, J. J. & Vanderloo, J. P. Vancomycin tolerance in methicillin-resistant *Staphylococcus aureus*: influence of vancomycin, daptomycin, and telavancin on differential resistance gene expression. *Antimicrobial agents and chemotherapy* **56**, 4422-4427, doi:10.1128/aac.00676-12 (2012).

4 Scherl, A. *et al.* Exploring glycopeptide-resistance in *Staphylococcus aureus*: a combined proteomics and transcriptomics approach for the identification of resistance-related markers. *BMC genomics* **7**, 296, doi:10.1186/1471-2164-7-296 (2006).
